# Cannabidiol in Neurology: Current Insights and Translational Perspectives

**DOI:** 10.3390/ph19020330

**Published:** 2026-02-17

**Authors:** Magdalena Białoń, Marta Kędziora, Katarzyna Starowicz

**Affiliations:** Department of Neurochemistry, Maj Institute of Pharmacology, Polish Academy of Sciences, 31-343 Cracow, Poland; bialon@if-pan.krakow.pl (M.B.); bryk@if-pan.krakow.pl (M.K.)

**Keywords:** cannabidiol (CBD), epilepsy, multiple sclerosis, neuropathic pain, Parkinson’s disease, Alzheimer’s disease, stroke, traumatic brain injury, nabiximols, Epidiolex

## Abstract

Cannabidiol (CBD) is one of the most studied compounds of *Cannabis sativa* and has attracted significant interest due to its therapeutic and beneficial properties, which have been confirmed in numerous preclinical and clinical studies over the last few years. A great advantage of CBD over the other widely known *Cannabis sativa* ingredient, Δ-9-tetrahydrocannabinol (THC), is that CBD does not exert intoxicating and psychoactive effects, making it an attractive candidate for therapeutic applications in neurological disorders. CBD has been shown to exert antioxidant, analgesic, anti-inflammatory, and neuroprotective effects, with therapeutic potential for various neurological conditions. To date, the only drug that consists solely of highly purified CBD is Epidiolex, which is used in the management of severe forms of epilepsy such as Dravet syndrome and Lennox–Gastaut syndrome. Another legal medication containing CBD (albeit with the addition of THC) is Sativex, used to alleviate spasticity in multiple sclerosis. Besides epilepsy, preclinical data suggest that CBD alone may be potentially beneficial in treating chronic pain, multiple sclerosis, Alzheimer’s and Parkinson’s diseases, or stroke. The safety profile of CBD is generally considered favorable, as the most commonly reported adverse effects are mild (e.g., somnolence, diarrhea). However, much attention should be paid as CBD-driven drug–drug interactions have been reported. This review article aims to assess the outcomes of preclinical and clinical research on CBD’s effects in various neurological conditions while also addressing potential risks and concerns related to its use.

## 1. Introduction

**Figure 1:**
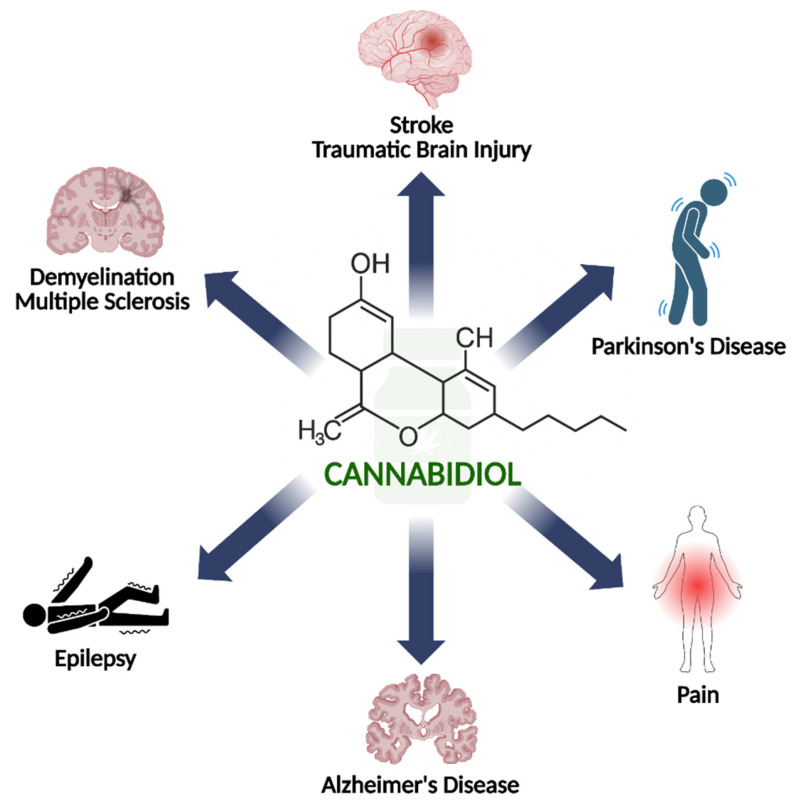
The application of cannabidiol in various neurological disorders reviewed in the article. Created in BioRender. Bialon, M. (2026) https://BioRender.com/t4plwbj.

Cannabidiol (CBD) is a phytocannabinoid that naturally occurs in the *Cannabis sativa* plant. In recent years, CBD has been intensively studied in preclinical models and clinical trials in order to determine its effectiveness in reducing symptoms of various diseases. These reports show that CBD can reduce pain and inflammation and improve behavioral conditions across different pathological states. The other best-known component of *Cannabis* is Δ-9-tetrahydrocannabinol (THC). However, THC causes psychoactive effects, which is why its use in the clinic is limited. In contrast to THC, CBD does not possess psychoactive properties and offers potential therapeutic benefits; it is now being extensively studied in other models of various disorders, both in vitro and in vivo. Preliminary studies show its effectiveness in diseases such as chronic pain, anxiety, and sleep disorders [1,2,3,4]. In the clinic, the most well-established and legally approved application of CBD is its use as an anticonvulsant in the treatment of epilepsy. The drug Epidiolex is approved in the United States to treat seizures associated with Lennox–Gastaut syndrome (LGS), Dravet syndrome (DS), or tuberous sclerosis in patients 1 year of age and older, whereas in Europe, it is approved from 2 years of age. The main aim of this article is to review the latest research on the effects of CBD in diseases such as epilepsy, multiple sclerosis (MS), Alzheimer’s disease (AD), stroke, traumatic brain injury (TBI), Parkinson’s disease (PD) and chronic pain (Figure 1) and summarize the latest discoveries and future directions in scientific research. This review is particularly aimed at translational researchers, as it integrates and critically evaluates preclinical and clinical evidence on CBD’s effects to assess its potential utility in the abovementioned neurological disorders.

## 2. Search Methodology

**Figure 2:**
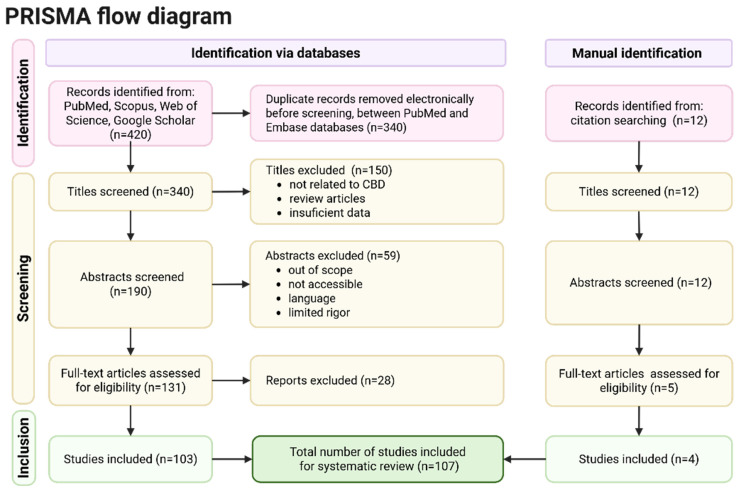
PRISMA flow diagram for study selection. The diagram represents the identification, screening, eligibility assessment, and inclusion of studies. A total of 420 records were identified from databases and 12 from citation searching. After removing duplicates and applying screening and eligibility criteria, 107 studies were included in the final systematic review. Created in BioRender. Bialon, M. (2026) https://BioRender.com/0iypn4x.

A comprehensive literature search was performed using PubMed, Scopus, Web of Science, and Google Scholar to identify relevant peer-reviewed publications available through December 2025. The search strategy used combinations of the following keywords: “cannabidiol”, “CBD”, “neurological disorders”, “epilepsy”, “Parkinson’s disease”, “Alzheimer’s disease”, “multiple sclerosis”, “traumatic brain injury”, and “stroke”. Searches were restricted to articles published in English. Additionally, the reference lists of eligible articles were manually screened to identify further relevant studies. Studies were considered eligible if they evaluated the effects of cannabidiol in in vitro experimental systems, animal models of neurological disorders, or human clinical populations. Both preclinical and clinical investigations were included to provide a comprehensive overview of the mechanisms of action and therapeutic potential of CBD in neurological conditions. Eligible publication types comprised original research articles, review papers, and clinical trial reports where relevant to the scope of this review. Studies were excluded if they focused exclusively on THC, investigated whole-plant cannabis without distinguishing the specific effects of CBD, or addressed non-neurological indications. Non-peer-reviewed sources and publications lacking direct relevance to neurological outcomes were also excluded (Figure 2).

## 3. Pharmacological Profile of CBD

The endocannabinoid system (ECS) consists of main endocannabinoids (anandamide (AEA) and 2-arachidonoylglycerol (2-AG)), cannabinoid receptors (CB1 and CB2), and enzymes responsible for its synthesis and degradation. THC—the main psychoactive component of *Cannabis sativa*—binds directly to the CB1 receptors in the central nervous system, and this profile of action is responsible for its psychoactive effects, such as anxiety, psychosis, impaired coordination and cognition [5]. CBD’s mechanism of action is less direct: it does not directly bind to CB1 or CB2 receptors with high affinity but modulates ECS activity and influences other non-cannabinoid receptors, such as serotonin receptors (mainly 5-HT1A) and transient receptor potential (TRP) channels, and is an antagonist of G protein-coupled receptor 55 (GPR55) [6,7,8]. This is why it does not produce psychoactive effects like THC. CBD also influences the ECS by inhibiting the enzyme fatty acid amide hydrolase (FAAH), which degrades AEA. As a result, the level of AEA in the synapse is elevated, and its signal is prolonged. CBD also acts as an antagonist of FK506 binding protein 5 (FKBP5), a protein in the immunophilin family that promotes inflammation by activating nuclear factor-kappa B (NF-κB) [9] and facilitating interactions of IκB kinase (IKK) subunits [10]. CBD directly binds to FKBP5, stabilizes it, and inhibits the assembly of the IKK complex and the activation of NF-κB. This action prevents the production of proinflammatory factors, such as nitric oxide (NO), interleukin 1β (IL-1β), interleukin 6 (IL-6), and tumor necrosis factor α (TNF-α), which are normally triggered by lipopolysaccharide (LPS)-induced NF-κB activity [11]. This broad spectrum of action for its diverse therapeutic and neuroprotective effects.

A critical aspect of CBD’s pharmacological profile is its complex interaction with G protein-coupled receptors (GPCRs), which goes beyond simple agonism or antagonism [12]. Recent perspectives highlight that CBD is a prominent example of a ligand that exhibits pathway selectivity, or ‘biased signaling’. For instance, at the 5-HT1A receptor (a key target for its anxiolytic and neuroprotective effects), CBD may preferentially activate specific intracellular cascades over others, such as favoring G-protein activation while showing minimal beta-arrestin recruitment. Furthermore, CBD’s role as a negative allosteric modulator of CB1 and CB2 receptors demonstrates its ability to alter receptor conformation and subsequent signaling output in the presence of endogenous cannabinoids. This complex GPCR-mediated signaling, including its antagonism of GPR55, suggests that the therapeutic outcomes of CBD are not solely a result of receptor occupancy but are causally linked to the selective activation of downstream pathways that regulate neuronal excitability and neuroinflammation.

CBD may be administered through various routes. In preclinical studies, the most common routes are intraperitoneal injection, oral gavage, inhalation, and subcutaneous injection. Clinically, CBD is usually administered orally in the form of oils, capsules, or edible products. CBD works the fastest when vaporized; this formula also offers the highest bioavailability, although it may cause pulmonary problems. Topical and transdermal formulations are used for localized conditions. The method of CBD delivery plays a crucial role in determining its absorption, metabolism, and therapeutic effectiveness; therefore, selecting the appropriate route is essential for both experimental studies and medical treatment. CBD is hydrophobic, leading to poor absorption, and the portion that is absorbed undergoes significant first-pass metabolism. Research shows that the bioavailability of CBD after oral administration is approximately 6% but can be increased fourfold when CBD is administered with a high-fat meal [13]. Other studies indicate that the oral bioavailability of CBD ranges from 9% to 13%. Compared to other routes of administration, the onset of action for an oral dose is slower, generally occurring within 30 min to 2 h [14]. Bioavailability following smoking is around 31% [15]. Moreover, the half-life is around 1.4–10.9 h after oromucosal spray, 2–5 days after chronic oral administration, 24 h after i.v., and 31 h after smoking [15]. In summary, CBD has low oral bioavailability, which can be improved by administration with high-fat food or by using alternative routes of administration [16]. First-pass hepatic metabolism and poor intestinal absorption are the main pharmacokinetic limitations of CBD. The comparative pharmacokinetic parameters of the various administration routes are summarized in Table 1.

Understanding the clinical variability of CBD requires a thorough examination of its human metabolism and pharmacokinetic profile. CBD has low oral bioavailability and undergoes extensive hepatic first-pass metabolism, primarily driven by CYP3A4 and CYP2C19 isoenzymes. This metabolic pathway generates several derivatives, most notably 7-hydroxy-CBD (7-OH-CBD) and 7-carboxy-CBD (7-COOH-CBD). It is important to note that 7-OH-CBD is a pharmacologically active metabolite with a potency comparable to, or in some assays exceeding, that of CBD itself [17]. Therefore, the in vivo effects of CBD administration are likely a cumulative result of both the parent compound and its active metabolites. The high inter-individual variability observed in clinical trials may be attributed to variations in metabolic rates, genetic polymorphisms of CYP enzymes, and the significant ‘food effect’ on absorption. Accounting for the contribution of active metabolites is essential for establishing accurate dose–response relationships in neurological applications.

Despite rarely reported side effects and generally being perceived as a safe substance (in contrast to THC), CBD can sometimes cause side effects such as liver toxicity, drowsiness, or gastrointestinal problems, or it may interact with other drugs. Most of CBD’s adverse effects are mild; however, CBD can interact with other drugs the patient is taking. It was found that CBD co-administered with other classes of drugs, such as clobazam and valproate, can cause serious adverse effects [18]. A meta-analysis of randomized clinical trials shows that most of CBD’s adverse effects occur in children. When studies in childhood epilepsy were excluded, the only adverse outcome associated with CBD treatment was diarrhea [19]. This suggests CBD is, in general, well-tolerated and has relatively few serious side effects. The toxicity of CBD mostly depends on its dose; in doses higher than recommended for use in humans, it may cause developmental toxicity, embryo mortality, neurotoxicity, hepatotoxicity, male reproductive system changes and spermatogenesis reduction, organ weight alterations, or hypotension [20]. Some meta-analyses show that CBD is highly effective in treating epilepsy; however, its effectiveness in other conditions is mixed or comparable to that of a placebo [21] (Table 2). These studies emphasize that the side effects of CBD are usually mild, but they can be serious in interactions with medications (e.g., hepatotoxicity).

## 4. Evidence in Specific Neurological Disorders

### 4.1. Epilepsy

Epilepsy is a neurological disorder defined by recurrent, spontaneous seizures and accompanied by a wide range of alterations, including molecular impairments and behavioral, psychological, and social disturbances [42]. The ECS is known to regulate excitatory/inhibitory balance within neuronal circuits, and alterations of ECS markers have been reported in both epileptic patients [43,44] and animal models of the disease [45,46,47,48]. For this reason, targeting the ECS represents a promising approach for developing novel therapeutics with anticonvulsant properties. The first anticonvulsant effects of *Cannabis* were noted in the late nineteenth century by British neurologists who observed decreased seizure frequency in epileptic subjects treated with Cannabis [49,50]. However, since the 1970–80s, CBD has remained understudied in terms of its anticonvulsant action. The research paper by Chiu and coworkers [51] was among the first published papers to investigate the antiepileptic effects of CBD in a rat model of epilepsy. They demonstrated that CBD exhibits significant anticonvulsant activity and, importantly, even at high doses, does not exacerbate the symptoms of focal epilepsy. Within years, the body of preclinical and clinical research on the effects of CBD in epilepsy grew, and it has expanded substantially over the past decades. Building on this growing evidence, several experimental studies have explored the cellular and molecular mechanisms underlying CBD’s anticonvulsant and neuroprotective actions in the CNS.

In the in vitro kainate-induced seizure model, CBD has been shown to exert a neuroprotective effect, as it reversed phagocytosis of damaged neurons and blocked the M0-to-M1 microglia transition via mechanisms mediated by Transient Receptor Potential Vanilloid 2 (TRPV2) and 5-HT1A receptors [52]. In electrophysiological studies, CBD (10 μM) was reported to reduce excitatory synaptic transmission at pyramidal cell synapses in a voltage-dependent manner and to enhance inhibitory synaptic potentials (IPSPs), independently of CB1 receptors [53]. Similar results have been reported by [54], showing that CBD can inhibit epileptiform activity in vitro, potentially in a CB1 receptor-independent manner. More recently, CBD has been shown to reduce seizure-like events (SLEs) in neocortical mouse slices by decreasing seizure amplitude and frequency [55]. Additionally, CBD reduces glutamate release from highly purified isolated nerve terminals [56], modulates synaptic transmission of the human cortex, and decreases the intrinsic excitability of human pyramidal neurons [57]. As CBD does not bind to N-methyl-D-aspartate (NMDA) or alpha-amino-3-hydroxy-5-methyl-4-isoxazolepropionic acid (AMPA) receptors [58], other indirect mechanisms are thought to mediate the decreased glutamatergic response. Interestingly, Song and coworkers proposed a novel, possible mechanism of anticonvulsant CBD’s action through the DEC2–SCN2A regulatory axis, a quite novel molecular pathway, by upregulating DEC2 expression and reinforcing its direct transcriptional inhibition of the sodium voltage-gated channel alpha subunit 2 (SCN2A), contributing to the suppression of excessive neuronal activity [59]. Notably, DEC2 is reported to be upregulated in hippocampal tissue of the temporal lobe epilepsy (TLE) mice model [60], which points to its possible role in regulating neuronal excitability.

Results from in vitro experiments have been further validated by in vivo preclinical studies and animal behavioral assessments. CBD demonstrates efficacy in various animal models, particularly those simulating drug-resistant pediatric epilepsies [61,62]. CBD displays both antiepileptiform and antiseizure properties in vivo, showing anticonvulsant effects in models of temporal lobe and partial seizures [63]. In the FeCl_3_-induced posttraumatic epilepsy rat model, Ma and coworkers reported reduced severity of seizures and brain damage in animals pretreated with CBD [64]. CBD treatment also led to a significant reduction in the atrophy and death of parvalbumin (PV) and cholecystokinin (CCK) expressing interneurons and improved morphological impairments of these cells observed in epileptic rats [53]. Chronic CBD administration induced anticonvulsant and antiepileptogenic effects in the Wistar Audiogenic Rat strain, which is sensitive and susceptible to audiogenic seizures [61,65]. These results, derived from in vitro and in vivo preclinical studies, are supported by clinical data showing that CBD has demonstrated significant efficacy in treating epilepsy symptoms. CBD has been proven to exert beneficial effects as an adjunctive treatment for refractory seizures associated with LGS, a severe form of epilepsy occurring in children. Randomized controlled trials have consistently demonstrated that highly purified, plant-derived CBD significantly reduces the frequency of drop seizures in LGS patients across all age groups, with retention rates showing efficacy is sustained over the long term [66,67,68]. It is also important that caregivers frequently report improvements in non-seizure outcomes, such as alertness and communication, suggesting much broader benefit beyond seizure control [68,69].

Years of research into the anticonvulsant effects of CBD culminated in the fast progress and development of the first—and, to date, the only—CBD-based drug for treatment-resistant epilepsy. The crucial results on CBD effectiveness and safety, in terms of seizures, have been published by Devinsky and coworkers [22,70], which resulted in the entry of Epidiolex, a plant-derived CBD product, on the market. This product was approved by the FDA in 2018 for the management of seizures associated with LGS and Dravet syndrome (DS). Randomized controlled trials and subsequent long-term open-label extensions have consistently demonstrated that Epidiolex, as an adjunctive CBD treatment, significantly reduces the frequency of atonic seizures in LGS and convulsive seizures in DS, with median seizure reductions ranging from 37 to 50% compared to placebo [22,69]. Furthermore, it has been indicated that treatment not only provides sustained seizure control over several years but is also associated with improvements in non-seizure outcomes, such as alertness, cognition, and behavior, which are critical to the overall quality of life for these patients and their caregivers [68]. Along with improvement in seizure control and the overall quality of patients’ life, adverse effects may be noted while on Epidiolex pharmacotherapy; the most common ones include somnolence, fatigue, skin rash and erythema, decreased appetite, diarrhea, and insomnia [22].

### 4.2. Multiple Sclerosis

Multiple sclerosis (MS) is a chronic autoimmune disease of the CNS in which inflammatory focal lesions cause demyelination, axonal loss, and glial scarring of nerve fibers, gradually leading to chronic disability [71]. While evidence of cannabis use for MS symptoms dates back decades, the first controlled clinical studies evaluating the therapeutic effects of cannabinoids, including CBD alone or CBD + THC combinations, for MS-related symptoms began to emerge in the early 2000s, based on previous observations from the 1980s.

In preclinical in vitro models of MS, CBD has demonstrated significant neuroprotective, anti-inflammatory, and remyelination-supporting properties [72,73]. Studies using glial and neuronal cultures have shown that CBD can attenuate microglial activation and oxidative stress, thereby reducing neuroinflammatory damage associated with demyelination [74]. Similarly, CBD derivatives have been shown to activate neuroprotective pathways, such as PP2A/B55α/HIF, contributing to cell survival and functional recovery in neural cells under inflammatory stress [75]. A broader review of cannabinoids in CNS pathological models confirms CBD’s capacity to modulate cytokine release, protect oligodendrocytes, and enhance remyelination, supporting processes [76,77]. Collectively, these findings suggest that in vitro CBD exposure mitigates several cellular mechanisms underlying MS pathology, positioning it as a promising candidate for translational neurotherapeutic development vivo and warrants further in vivo animal research.

In animal-modeled studies of the disease, CBD alone or in combination with THC has been evaluated in terms of its efficacy in alleviating MS symptoms [78]. This line of evidence suggests that CBD administered at a low dose (5–20 mg/kg) significantly improves the scores of MS clinical signs in an Experimental Autoimmune Encephalomyelitis (EAE) murine model of the disease [79,80,81,82] and reduces demyelination and axonal damage [80,81,82,83]. This therapeutic effect is strongly linked to CBD’s potent anti-inflammatory and immunosuppressive properties. Recent studies in EAE mice confirm that CBD mitigates neuroinflammation by reducing leukocyte recruitment to the spinal cord and decreasing mRNA expression of adhesion molecules such as ICAM-1 and VCAM-1 [84], proteins crucial for immune and inflammatory processes. Furthermore, CBD treatment attenuated EAE hallmarks by promoting the induction of immunosuppressive myeloid-derived suppressor cells (MDSCs) and suppressing proinflammatory T cell responses, thereby decreasing cytokines such as interleukin 17 (IL-17) and interferon ɣ (IFN-ɣ) [80,81]. While CBD alone shows significant promise, the combination of THC and CBD has also revealed synergistic effects. Al-Ghezi and co-workers demonstrated that THC + CBD combined therapy (10 mg/kg each), but not THC or CBD alone, attenuated murine EAE by reducing neuroinflammation and suppressing Th17 and Th1 cells, and these effects were mediated by CB1 and CB2 receptors [85]. Additionally, CBD attenuated EAE and suppressed neuroinflammation by preventing microbial dysbiosis observed during the course of the illness [86]. This demonstrated synergistic action between THC and CBD in preclinical models of MS provides a strong rationale for the use of combination therapies in patients, a strategy that has already translated into a widely used clinical product such as nabiximols (Sativex^®^), which is one of the first cannabis-based medicines that has been approved as a prescription medicine used to alleviate MS-related neuropathic pain and spasticity. Sativex^®^ contains a strictly defined quantity of THC and CBD (27 mg/mL and 25 mg/mL, respectively) [87]. Clinical trials have demonstrated its efficacy in reducing patient-reported spasticity scores compared with placebo, with generally mild-to-moderate adverse effects, including dizziness, fatigue, and somnolence [88,89]. Randomized controlled clinical trials on Sativex have consistently demonstrated both short- and long-term efficacy in managing resistant spasticity associated with MS. Russo et al. [90] presented data showing that one-month continuous treatment with Sativex reduced pain, spasticity, and the number of daily spasm episodes and improved ambulation in MS patients. It has been hypothesized that the therapeutic action of nabiximols may be mediated through modulation of intracortical and spinal excitability [90]. Importantly, lines of evidence suggest that Sativex does not exert abuse potential in cannabis-naïve MS subjects [91]. These findings have been supported by a 48-week, double-blind, placebo-controlled study evaluating the effect of the drug on mood and cognitive functions in subjects experiencing spasticity related to MS [92]. However, Schoedel and coworkers [93] published a single-dose, randomized, double-blind, crossover study in which nabiximols exerted some addiction potential measured after a single administration, but only in higher doses. It should be emphasized that prolonged administration of Sativex at therapeutic doses is not associated with dose escalation, misuse, or abuse in patients [94,95]. Importantly, Sativex does not exert the side effects usually associated with recreational cannabis use and does not present long-term safety concerns, as it has not been associated with the development of drug tolerance or withdrawal, nor has evidence of misuse or abuse been reported. In the end, no evidence of THC or CBD accumulation in patients receiving self-titrated doses of combined cannabinoids has been reported [96].

### 4.3. Alzheimer’s Disease and Dementia

Progressive cognitive decline represents a hallmark feature of Alzheimer’s disease (AD) and has been mechanistically linked to amyloid β-protein (Aβ) deposition, tau pathology, neuronal loss, and neuroinflammatory processes [97]. Robust inflammatory responses have been consistently documented in both animal models of AD and in post-mortem brain tissue from affected individuals [98,99,100]. Given the anti-inflammatory and neuroprotective properties of CBD, this compound has emerged as a promising candidate for therapeutic intervention in AD. As shown by Mello-Hortega and colleagues [101], studies have mostly focused on amyloid-β pathology, behavioral evaluation, neuroinflammation, oxidative stress, and physiological changes observed in AD.

As previously shown, neuronal death is linked to memory decline in AD [102]. Therefore, the effects of CBD on cellular death in in vitro models have been evaluated to better understand its effects and the mechanism responsible for its protective function. Raich and coworkers [103] reported that CBD stimulation resulted in decreased aggregation of pTau and amyloid-β, reducing IL-1β and increasing interleukin 10 (IL-10) expression in mice microglial primary cultures. These results are consistent with other findings confirming CBD’s neuroprotective role, as it improved cell viability and decreased lipid peroxidation and oxidative stress in hippocampal cells [104]. In a similar context, CBD reduced both inducible nitric oxide synthase (iNOS) protein expression and nitrite production triggered by amyloid-β (Aβ_1–42_) stimulation in P12 cells. This response occurred in a concentration-dependent manner and has been associated with the inhibition of phosphorylated p38 mitogen-activated protein kinase (MAPK) and the downregulation of nuclear factor-κB (NF-κB) activation [105]. Additionally, CBD prevents tau aggregation [106], enhances neurogenesis, and stabilizes redox balance by modulating antioxidant enzymes, such as superoxide dismutase (SOD) and catalase (CAT) [107]. Recent studies further support the notion that CBD-loaded nanocarriers improve neuronal uptake and enhance their antioxidative efficacy in hippocampal cultures [108,109]. These findings underscore CBD’s promise as a multifaceted neuroprotective agent in AD pathophysiology, primarily by counteracting oxidative and inflammatory responses at the cellular level in hippocampal cells. Findings from in vitro models are further corroborated by animal studies, in which CBD demonstrates comparable benefits, translating cellular mechanisms into improved cognitive and behavioral outcomes in AD models. These converging lines of evidence suggest that the molecular effects observed in cultured neurons are consistent with the systemic neuroprotective benefits seen in vivo. In a rat model of streptozotocin-induced AD features, chronic CBD treatment restored behavioral deficits, reduced neuroinflammatory markers expression, and mitigated AD-associated changes [97]; it improved both short- and long-term memory parameters assessed in the novel object recognition (NOR) test, and this phenomenon was accompanied by improved glucose metabolism [110]. In mouse models of AD, CBD improved cognitive deficits, including memory and learning impairments [103,111,112,113]. Additionally, CBD has been reported to ameliorate anxiety symptoms in AD-modeled mice [111], which stands for another favorable effect of CBD in the course of the disease, as anxiety symptoms occur in ~40% of AD patients [114]. These behaviorally beneficial outcomes may be related to several molecular effects. CBD reduced oxidative stress parameters (SOD1, SOD2, 4-HNE, and gp91pho) in the hippocampal region [111] and promoted microglia shift from a pro- to an anti-inflammatory state [103,112]. Furthermore, CBD treatment upregulated interleukin-33 (IL-33) and triggering receptor expressed on myeloid cells 2 (TREM2), molecular factors likely associated with improvements in neurological function [115]. Interestingly, RNA sequencing of blood and brain samples from AD mice revealed that more than 75% of AD onset markers (among more than 1000) were eliminated or reversed in response to dietary CBD [116]. However, previously published studies have shown contradictory effects of CBD treatment on Aβ40 concentrations, ranging from the ability to reduce protein levels or aggregation [103,117,118] to slight or no effect [113,119,120,121].

Given the demonstrated neuroprotective and antioxidative properties of CBD alone, which may be particularly advantageous in the context of AD progression, several studies have sought to evaluate the therapeutic potential of combined THC and CBD treatments as a more effective intervention. CBD and THC co-administration improved spatial memory, anxiety, and depressive behavior [122], reduced basal excitability (but not synaptic plasticity), and reversed glutamate uptake deficit in the hippocampus of AD-modeled mice [123]. However, in the study by Aumer and colleagues [124], CBD combined with THC did not improve any of the analyzed parameters, disrupted performance in the NORT, and exerted an opposite effect in both AD-modeled and control mice, as measured in the elevated plus maze, suggesting genotype-dependent effects. While preclinical studies have demonstrated that CBD alone can reduce neuroinflammation and oxidative stress [125], CBD and THC combinations exert synergistic effects, improving cognitive function and modulating amyloid and glutamate pathways [126]. Recent findings suggest that dual cannabinoid formulations may offer superior neuroprotective and anti-amyloid benefits compared to single-compound treatments.

Clinical trials investigating CBD for AD and dementia primarily focus on managing behavioral and psychological symptoms, with less emphasis on the disease-modifying effects observed in preclinical models [127,128]. Current evidence, derived from both randomized controlled trials and prospective open-label studies, suggests that CBD cannabis extracts are generally well-tolerated and offer a promising therapy for these symptoms [129,130]. A placebo-controlled randomized controlled trial found that a CBD-dominant oil significantly reduced agitation compared to placebo in patients with dementia-related behavioral disturbances [34]. Furthermore, a recent open-label prospective cohort study showed that treatment with CBD-rich oil in AD patients resulted in a significant and sustained reduction in overall Neuropsychiatric Inventory-Questionnaire (NPI-Q) severity and caregiver distress scores over a 24-month follow-up period [131]. While these results are encouraging for symptomatic management, larger, long-term RCTs are still needed to establish optimal dosing, long-term safety, and any potential effects of CBD on cognitive decline or disease progression itself [128,129].

### 4.4. Parkinson’s Disease

PD is a degenerative brain disorder involving the gradual destruction of dopamine-secreting cells in the substantia nigra pars compacta, causing reduced dopamine levels in the striatum. The disease presents clinically with four primary motor features: tremor at rest, slowness of movement, muscle stiffness, and balance difficulties, accompanied by various non-motor symptoms, including cognitive decline or sleep disorders. Despite advances in symptomatic pharmacotherapy, such as L-DOPA, the progression of neurodegeneration underscores the urgent need for disease-modifying interventions.

The potential of CBD in PD management stems from its multimodal pharmacological profile, which addresses both the underlying neurodegenerative process and symptomatic complications. Evidence for the efficacy of CBD in PD primarily comes from preclinical models, where CBD exhibits neuroprotective and symptomatic effects. The neuroprotective role of CBD is primarily mediated through the attenuation of oxidative stress and neuroinflammation. Clinical studies are limited, yielding mixed results. Some studies indicate improvements in both motor and non-motor symptoms [132], while others do not confirm benefits over placebo [36]. In an in vitro study on the PC12 cell line exposed to 6-hydroxydopamine (6-OHDA), CBD augmented cell viability and decreased apoptosis. Mechanistically, CBD has been shown to activate the Nrf2 pathway, leading to increased antioxidant expression (e.g., Bcl-2) and reduced pro-apoptotic markers (e.g., Casp3, Bax) [133]. In a rat model of PD, chronic treatment with CBD reduced nigrostriatal degeneration and neuroinflammation while improving motor performance. The mechanism of CBD’s action was shown to involve activation of astrocytic TRPV1 receptors and enhancement of the endogenous neuroprotective response to ciliary neurotrophic factor (CNTF) [134]. In a mouse model of PD, CBD (both acute and chronic treatment) reduced hyperalgesia and allodynia evoked by 6-OHDA. What is more, while CBD was administered at ineffective doses of either an FAAH inhibitor or a TRPV1 receptor antagonist, its effectiveness was potentiated [135]. Furthermore, CBD appears to modulate the complex signaling network involved in motor control. In various animal models (6-OHDA, reserpine, and rotenone), CBD administration consistently reduces α-synuclein accumulation, preserves tyrosine hydroxylase-positive neurons, and delays the onset of motor deficits [136,137]. A prolonged administration of L-DOPA (a gold standard in PD treatment) may lead to L-DOPA-induced dyskinesia (LID). Nascimento et al. found that CBD and PECS-101 (a fluorinated CBD derivative) reduced abnormal involuntary movements without impairing the motor benefits of L-DOPA, and the effect was prevented by CB1 and PPARγ receptors antagonists, while capsazepine (TRPV-1 antagonist) enhanced the antidyskinetic effects of CBD [138]. A promising new approach to the use of CBD in the treatment of PD is the use of cannabidiol lipid nanoparticles. Lapmanee et al. demonstrated that CBD lipid nanoparticles administered to PD rats reduced lipid profiles, enhanced insulin secretion, and restored dopamine levels, compared with CBD in its natural form, and had comparable effectiveness to L-DOPA in ameliorating motor deficits and memory impairment [139].

However, a significant translational gap exists between these robust preclinical findings and the results of clinical trials, which remain largely equivocal. While some studies suggest improvements in quality of life, anxiety, and tremor amplitude during specific tasks [37,39], larger randomized controlled trials have failed to demonstrate superior motor or cognitive benefits over placebo [36,38]. For example, PD patients who received high CBD/low THC extract experienced no benefits, worsened cognition and sleep, and many mild adverse events in the treated group compared to placebo [137]. This discrepancy may be attributed to several factors. Firstly, traditional oral delivery of CBD suffers from low bioavailability. Emerging strategies, such as lipid nanoparticles, have shown promise in animal models by restoring dopamine levels more effectively than free CBD, suggesting that suboptimal delivery may be a hurdle in human trials [139]. Moreover, clinical protocols vary significantly in dosage and duration, complicating the identification of a therapeutic window. Finally, most clinical failures use pure CBD isolates, whereas preclinical successes often hint at synergistic interactions within the endocannabinoid system, such as FAAH inhibition or TRPV1 antagonism [135].

In summary, preclinical studies using CBD in in vitro and animal models have yielded promising results. However, clinical trials in PD patients show that CBD does not have such direct positive effects in the treatment of PD. However, clinical studies differ in terms of CBD dosage and treatment duration, so more research is needed to clearly determine CBD’s effectiveness. Future research should prioritize high-bioavailability formulations and focus on specific patient subpopulations, particularly those suffering from non-motor symptoms or L-DOPA-induced complications.

### 4.5. Stroke and Traumatic Brain Injury

TBI is a complex injury that triggers neuroinflammation, glial cell activation, and cell death. This is followed by the later onset of severe psychological symptoms and cognitive impairments. TBI most significantly and lastingly affects a person’s neurological function, but it may also be associated with chronic pain, anxiety, and depression. Current therapeutic options to treat TBI and reverse its undesirable effects are limited. Similarly, treating stroke is a big challenge in medicine since there are no highly effective therapies available. The results of CBD’s neuroprotective effects in stroke and traumatic brain injury are limited, but indicate the potential neuroprotective effects of CBD, mostly via modulation of neuroinflammation, including glial activation.

Belardo et al. found that mice with TBI developed chronic pain, anxious/aggressive behaviors, depressive-like behavior, and impaired social interaction. Moreover, TBI mice showed altered neurotransmitter release in the cortex. Oral administration of 10% CBD oil from day 1 to day 14 and from day 50 to day 60 reversed the behavioral changes and partially normalized the cortical biochemical changes [140]. In stroke treatment, intraperitoneal CBD treatment prevented ischemia-induced neurological impairment and reduced microglial activation and the neurological deficit score in mice, which indicates that the neuroprotective effects of CBD may occur in the subacute phase of ischemia [141]. In a mouse stroke model, CBD was proven to ameliorate mitochondrial dysfunction and attenuate neuronal injury in rats following cerebral ischemia [142]. Also, CBD reduces production of IL-1β and TNF-α and microglial activation; ameliorates mitochondrial deficits; and decreases NFκB phosphorylation in BV-2 cells subjected to oxygen-glucose deprivation/reoxygenation. The effect was cyclin-dependent kinase regulatory subunit 1B (CKS1B)-dependent, which suggests that CKS1B is a regulator of neuroinflammation and is involved in the anti-inflammatory effects of CBD [143]. Dong et al. showed that CBD ameliorated motor, memory, and cognitive functions in TBI mice and reduced the concentration of phosphorylated tau protein and amyloid-β [144]. Oral CBD pretreatment before TBI reduced TBI-induced glutamate release (as measured by cortical microdialysis) and improved sensorimotor function, facilitating the animal’s functional recovery [145].

### 4.6. Neuropathic Pain

Recent experimental animal studies have confirmed that CBD (both in its pure form and as a component of hemp extracts) relieves neuropathic pain. Its effects are not limited to the ECS. CBD has also been shown to have anti-inflammatory properties, alter nerve cell activity, and affect other, lesser-known pain pathways. For example, Wang et al. showed that oral CBD administration attenuates neuropathic pain and chronic constriction injury (CCI)-induced microglia activation and FKBP5 overexpression in the lumbar spinal cord dorsal horn [11]. In the cisplatin-induced mouse model of neuropathic pain, pure CBD had little impact on mechanical hypersensitivity, in contrast to THC, which had an analgesic effect. The treatment of neuropathic pain is difficult, and patients are often forced to take strong medications. Unlike morphine, CBD does not induce pharmacological tolerance over time, and it exhibits a significant opioid-sparing effect, potentially allowing for lower morphine doses to achieve adequate relief [146,147]. However, a significant ‘potency gap’ exists when comparing pure CBD to THC-containing formulations. While pure CBD often shows modest results in clinical settings for conditions, high-CBD cannabis extracts or THC/CBD combinations (such as Sativex) often demonstrate superior efficacy [148,149]. THC has an analgesic effect, but it also causes several side effects, while CBD does not cause any undesirable side effects but has a weaker effect than THC in reducing allodynia. A combination of THC and CBD can produce a dose-dependent reduction in allodynia; however, it displays little to no synergy. A key finding emerging from preclinical models is the distinction between preventive and therapeutic windows. For instance, in chemotherapy-induced neuropathy (paclitaxel model), CBD and its analogs can successfully prevent the development of mechanical sensitivity, yet they often fail to reverse established, chronic allodynia [150]. This suggests that CBD’s primary strength may lie in its neuroprotective capacity to inhibit the transition to chronic pain states rather than in the acute suppression of existing pain signals. CBD’s prevention of paclitaxel-induced neuropathy was blocked by AM630 (CB2 receptor antagonist). Moreover, CBD inhibits spinal expression of toll-like receptor 4 (TLR4) and onized calcium-binding adapter molecule 1 (Iba1), increases spinal levels of 2-AG and AEA, and reduces cytokine levels in mice with neuropathic pain [151]. Boccella et al. proved that one-week CBD treatment can reverse changes triggered by spared nerve injury (SNI)-induced neuropathic pain, such as allodynia, an increase in dynorphin peptide and its kappa opioid receptors (KORs) in the hippocampus’ dentate gyrus (DG), memory deficits, LTP impairment in the entorhinal cortex-DG, downregulation of 2-AG, and upregulation of the cannabinoid CB1 receptors in the DG [152]. A clinical study on 29 patients with symptomatic peripheral neuropathy showed that four weeks of CBD treatment (50 mg CBD/3 fl. oz) significantly reduced intense pain, sharp pain, and cold and itchy sensations, with no adverse effects [40]. A systematic review of 927 studies showed that cannabis-based medicines may be effective in treating the pain and symptoms of peripheral neuropathy, compared to a placebo [153]. Other clinical trials tested the VER-01 product, consisting of unfragmented, dried *Cannabis sativa* DKJ127 L. flowers. Chronic low back pain patients who took VER-01 for 12 weeks experienced reduced pain compared to the placebo group; the compound was also well-tolerated with no signs of dependence or withdrawal [41].

However, there are also experimental studies that indicate no or limited efficacy of CBD in alleviating neuropathic pain in specific models or conditions, e.g., in the case of already developed allodynia or in specific models of neuropathy. As mentioned previously, CBD can prevent the development of mechanical sensitivity in chemotherapy-induced neuropathy but may not reverse existing mechanical allodynia, indicating a lack of analgesic effect in established neuropathic pain [150]. Moreover, a clinical study of CBD’s effectiveness in patients with hand osteoarthritis or psoriatic arthritis showed that synthetic CBD (20–30 mg) administered daily for 12 weeks did not significantly affect pain intensity, sleep quality, depression, anxiety, or pain catastrophizing scores compared with placebo [154]. Also, an 8-week treatment with CBD failed to reduce pain in patients with painful polyneuropathy, post-herpetic neuralgia, and peripheral nerve injury. In the same study, it was also shown that neither THC nor the THC + CBD combination had any effect [155]. These results indicate that despite positive reports from preclinical studies, the effectiveness of CBD is limited in the treatment of chronic pain.

Despite these promising mechanisms, clinical data for pure CBD in chronic pain remain limited and frequently contradictory. While some small-scale studies report significant relief in peripheral neuropathy [40,41], larger trials in osteoarthritis and mixed chronic pain populations have failed to outperform placebos [154,155]. These discrepancies may stem from the lack of standardized dosing, the timing of intervention (preventive vs. late-stage), and the inherent difficulty of treating advanced neuropathic states with non-psychoactive monotherapy. Future clinical efforts should therefore focus on high-bioavailability formulations and early-intervention strategies to fully leverage CBD’s neuroprotective potential.

## 5. Challenges and Future Directions

CBD has been tested in various diseases, but its effectiveness appears to be limited. The difficulty with using CBD is the variety of forms of administration and doses used. There are several delivery methods: inhalation (the fastest, with the highest absorption), oral delivery (the slowest, with the lowest absorption), and sublingual, topical, and transdermal delivery. The legal status of therapeutic CBD products is not consistent globally and varies by country. Generally, products containing less than 0.3 or 0.2% THC are legal in most countries in Europe and in the US. Depending on the country, CBD sales must comply with local regulatory bodies and are usually classified as a medicine, food/dietary supplement, or cosmetic. It is worth noting that dietary supplements are not subject to as strict restrictions and controls as medicines are, so the quality and purity of the CBD sold should be taken into account.

The CBD’s effectiveness has been best researched and well-documented in the treatment of epilepsy. The drug Epidiolex is registered and successfully used in many countries around the world, including in children. However, the effectiveness of CBD in other disorders varies and depends on the dose and duration of use. For example, some promising reports suggest that CBD and THC might be effective in treating Huntington’s disease [156,157]. However, other published studies on Huntington’s disease indicate no or limited effectiveness of CBD [158,159]. Clinical studies showed that CBD (Sativex) is safe and well-tolerated in patients with Huntington’s disease, with no worsening of symptoms, but no significant differences in motor or cognitive scales were observed vs. placebo [160]. Also, as mentioned above, clinical trials of CBD differ from those from preclinical studies in animal models. In cancer patients, no detectable effect of CBD on quality of life, depression, or anxiety was found, even if patients reported feeling better; a similar improvement in well-being was seen in the placebo group [161,162]. In breast cancer patients with taxane-induced peripheral neuropathy, CBD was not effective in reducing pain and improving functional well-being, and in addition, it worsened ratings of sleep [163].

Another challenge associated with the use of CBD in clinical research is the difficulty in separating its pharmacological outcomes from placebo and expectancy effects. Clinical trials evaluating CBD’s effectiveness in various conditions, e.g., anxiety and stress, often fail to demonstrate benefits of CBD beyond placebo. In a randomized trial testing low-dose over-the-counter CBD oil for perceived stress and psychological distress, both the CBD and placebo groups showed similar improvements, with no significant differences between groups, suggesting a strong placebo effect on the observed outcomes [164]. Additionally, previous research indicates that expectancy (believing that one has taken CBD) can affect subjective stress and anxiety responses in the laboratory setting, even when no active CBD is present in the treatment [165]. Cannabinoids can produce recognizable sensory or physiological effects (e.g., changes in alertness, dizziness, dry mouth), allowing participants, and sometimes investigators, to infer treatment allocation. While this issue is more evident with THC-containing products, it may also occur with higher doses of CBD. Imperfect blinding increases the risk of performance and detection bias, potentially inflating efficacy estimates or altering adverse event reporting. Another important issue that should be emphasized is that CBD can interact with many drugs and substances [166], mainly by inhibiting cytochrome P450 (CYP450) enzymes in the liver, since CBD is metabolized by CYP3A4 and CYP2C19 enzymes [167]. Recent research has shown that CBD selectively inhibits CYP2C19 and CYP3A4 by competitive binding, affecting the metabolism of drugs that are substrates for these enzymes (see review [168]). Additionally, CBD acts as a potent inhibitor of p-glycoprotein (P-gp), the major efflux transporter expressed in the liver, kidneys, intestines, and BBB [169], and plays a key role in reducing drug uptake and enhancing its clearance. At high concentrations, CBD may significantly influence the absorption and metabolism of drugs that are P-gp substrates [170], such as antipsychotic drugs like olanzapine and risperidone [171]. Such interactions may alter drug levels in the bloodstream, potentially increasing the risk of side effects or compromising the drug’s intended benefits. The effects depend on the CBD dose, route of administration, and the patient’s condition. Due to CBD’s greatest effectiveness in treating epilepsy, it may interact with anticonvulsant drugs such as clobazam by increasing the concentration of drugs and their metabolites [172] and enhancing clobazam’s sedative effects. Therefore, when introducing CBD to patients already using clobazam, it is crucial to monitor symptoms of drug toxicity. CBD has also been reported to interact with another antiepileptic drug, valproate, leading to increased liver transaminase levels [70]. Regarding pain pharmacotherapy, CBD may potentially inhibit UGT2B7 activity, thus enhancing the level and effect of morphine [173,174]. Moreover, CBD affects the metabolism of antidepressants such as citalopram, fluoxetine, amitriptyline, or mirtazapine by inhibiting the CYP enzyme family, leading to an exacerbation of drugs’ side effects [175]. CBD’s interaction with other therapeutics, such as anticoagulants or immunosuppressant drugs, is also probable [167]. Recent studies have demonstrated associations between CYP2C9, CYP2C19, and CYP3A4/5 activity and genotypes, CBD metabolism, and the occurrence of adverse effects [17,176], suggesting that pharmacogenetic profiling may represent a promising tool for assessing and predicting the responsiveness, clinical utility, and effectiveness of CBD in specific patient populations. This approach is particularly relevant in the context of polypharmacy, where CBD may increase the risk of clinically significant cytochrome P450-mediated drug–drug interactions.

From a pharmacokinetic perspective, CBD might be a significant challenge. Its bioavailability after oral administration is very low (estimated at 6–10%) and highly sensitive to the “food effect,” in which high-fat meals can increase absorption several times. Combined with individual differences in CYP450 enzyme activity, this leads to irregular plasma levels, making it a major challenge to standardize doses across different patient groups. Substantial heterogeneity in cannabinoid formulations further limits comparability across studies. Clinical trials differ widely in formulation type (purified CBD vs. full-spectrum extracts or CBD/THC combinations), route of administration, dosing regimens, and titration protocols. These differences strongly influence pharmacokinetics, including bioavailability, peak plasma concentrations, and interindividual variability. Low and variable oral bioavailability, extensive first-pass metabolism, and food effects complicate dose–exposure–response relationships, making cross-trial comparisons challenging.

Additional variability arises from differences in formulation quality, excipients, and stability, even among regulated products. Such factors may affect both efficacy and tolerability, hindering the derivation of consistent dosing recommendations. Overall, these limitations highlight the need for improved trial design in cannabinoid research, including more robust blinding strategies, standardized formulations, and pharmacokinetic-informed dosing approaches to enhance reproducibility and translational relevance.

## 6. Conclusions

CBD has established effectiveness in managing specific severe forms of epilepsy, such as Dravet and Lennox–Gastaut syndromes, and is successfully applied in medical practice for these disorders. However, for other diseases, including MS, AD, PD, stroke, or chronic pain, the evidence is still preliminary. Although encouraging findings have emerged from preclinical research, results from clinical trials are frequently mixed, inconclusive, or limited in scope. In conclusion, more rigorous, large-scale human research is critically needed to either confirm the effectiveness of CBD in these other areas or definitively exclude its therapeutic use.

Moreover, CBD has experienced a significant increase in commercial popularity that currently far exceeds the robust data from large-scale randomized controlled trials for most of its proposed neurological and psychiatric indications. The largest current clinical interest surrounding CBD involves its use for anxiety disorders, insomnia, and pain management; the strongest clinical evidence for CBD is currently restricted to its use in certain forms of epilepsy. A major issue is the regulatory status of many commercially available CBD products, which are often sold as dietary supplements. This status raises significant concerns about product quality and safety. A major safety risk is the presence of THC at levels that exceed the declared limit, which can lead to unwanted psychoactive effects and legal issues.

Future research should focus on innovative delivery systems, e.g., utilizing nanoemulsions or lipid nanoparticles to improve bioavailability and reduce pharmacokinetic variability; developing more sophisticated placebos that mimic the sensory and side-effect profile of CBD; and identifying objective neuroimaging or biochemical markers to measure response, thus reducing our reliance on subjective reporting.

To sum up, there is an urgent need for robust translational research. The next critical step involves moving toward a precision medicine approach, seeking novel biomarkers, validating specific targets that predict a therapeutic response to CBD, and patient profiling to ensure treatment is targeted, effective, and safe.

## Figures and Tables

**Table 1:** Comparative pharmacokinetic parameters for various administration routes of CBD.

Route of Administration	Bioavailability (%)	Onset of Action	Half-Life (t1/2)	Key Characteristics
Oral (Capsules/Oil)	~6–13%	30 min–2 h	2–5 days (chronic)	Subject to extensive first-pass metabolism; 4× increase in absorption with high-fat meals
Inhalation (Smoking/ Vaporization)	~31%	Rapid (s/min)	~31 h	Highest bioavailability; bypasses first-pass metabolism; potential pulmonary risks
Intravenous (i.v.)	100%	Immediate	~24 h	Used primarily in clinical research settings

**Table 2:** Overview of clinical studies on CBD use in neurological disorders.

Disease	Type of Study	Administered Compounds and Dosage	Subjects	Time	Endpoint/Outcomes	Results	Reference
Dravet Syndrome (DS)	multinational, randomized, placebo-controlled double-blind trial	purified cannabidiol (CBD) 20 mg/kg/day or placebo, in addition to standard antiepileptic treatment	120 children and young adults	4-week baseline period, a 14-week treatment period	change in convulsive seizure frequency over a 14-week treatment period, as compared with a 4-week baseline period	positive	[22]
Lennox–Gastaut syndrome (LGS)	randomized, double-blind, placebo-controlled trial	20 mg/kg/day oral purified CBD or placebo	171 patients receiving CBD (n = 86) or placebo (n = 85)	14 weeks	percentage change from baseline in the monthly frequency of drop seizures during the treatment period	positive	[23]
LGS	double-blind, placebo-controlled trial	purified CBD 10 mg/kg/day, 20 mg/kg/day, or placebo	293 patients were assessed; 68 were excluded	4-week baseline period + 14-week treatment period	The primary outcome was the percentage change from baseline in the frequency of drop seizures (average per 28 days)	positive	[24]
LGS	open-label extension trial	Epidiolex (100 mg/mL), titrated from 2.5 to 20 mg/kg/day; addition to existing antiepileptic drugs	treatment was ongoing in 299 patients, age 2–55 years old, 54% M; 46% F; 208 patients completed 48 weeks of treatment	median treatment duration: 263 days (38 weeks; range 3–430 days),	evaluation of the long-term safety and tolerability of adjunctive CBD treatment	positive	[25]
Drug-resistant focal epilepsy	randomized, double-blind, placebo-controlled multicenter clinical trial	195 mg (approximately 2.6 mg/kg) or 390 mg (approximately 5.3 mg/kg) transdermal purified CBD or placebo; twice daily	188 patients (85 M; 103 F), age: 18–70 years old	12 weeks	least squares mean difference in the log-transformed total seizure frequency per 28-day period, adjusted to a common baseline log seizure rate, during the 12-week treatment period	negative	[26]
Drug-resistant epilepsy	prospective, open-label cohort study	purified CBD as an adjunct anti-epileptic drug, titrated to a maximum of 25 mg/kg/day	22 M and F were enrolled; mean age: 8.4 years old; 36 completed 12 weeks’ therapy	12 weeks	evaluation of the tolerability and safety of CBD for treating drug-resistant epilepsy in children, and to describe adverse events associated with such treatment	positive	[27]
Multiple sclerosis (MS) spasticity	randomized controlled trial	Sativex (CBD + THC); subjects were instructed to titrate their daily dose steadily as required over 2 weeks, to a maximum of 48 sprays per day	189 subjects were randomized (124 to Sativex, and 65 to placebo); subjects over 18 years old, 75 M, 114 F	6 weeks	change from baseline in the severity of spasticity based on a daily diary assessment by the subject on a 0–10 numerical rating scale (NRS)	positive	[28]
MS spasticity	a double-blind, placebo-controlled, randomized clinical trial	Sativex (CBD + THC); patients up-titrated the dosage of spray to a maximum of 12 sprays/day or placebo	subjects over 18 years old; Phase B: 109 patients; for placebo (n = 46), Sativex (n = 48)	12 weeks phase B	≥30% improvement of the numerical rating scale (NRS) of spasticity	positive	[29]
MS spasticity	phase 3, randomized, double-blind, placebo-controlled crossover trial	Sativex (CBD + THC); treatment periods consisted of a 14-day dose titration phase, in which patients were advised to titrate their dose beginning with 1 spray/day to an individually optimized dose, up to a maximum of 12 sprays/day	68 patients	21 + 21 days	change in velocity-dependent muscle tone as measured by the Modified Ashworth Scale (MAS) Lower Limb Muscle Tone-6 from day 1 predose to day 21 (period 1) and from day 31 predose to day 51 (period 2)	the primary endpoint was not met	[30]
MS spasticity	double-blinded clinical trial	purified CBD oral drops; initially 5 mg/day, increasing to 70 mg/day over 2 weeks, and 80 mg/day from the third week to the fourth week	49 MS patients; CBD (n = 24) or placebo (n = 25); the mean age: 40.65 ± 7.35 years old	8 weeks (4 weeks of treatment and 4 weeks of follow-up)	spasticity reduction measured in T25-FW test	mixed	[31]
Alzheimer’s disease (AD)	randomized, double-blind, placebo-controlled trial	oral capsules of purified CBD (200 mg) or placebo, starting with one capsule/day and titrated upwards to 3 capsules/day;	patients with AD and with behavioral and psychological symptoms of dementia; CBD (n = 8) and placebo (n = 7), mean age: 77.91 ± 8.08 years old	6 weeks	the primary endpoints were acceptability, adherence to treatment, and retention rates from baseline to week 6, while secondary outcomes included safety/tolerability and clinical and cognitive measures	according to the primary endpoints: positive	[32]
AD	randomized, double-blind, placebo-controlled feasibility trial	Sativex (CBD + THC); The target dose was four sprays/day of nabiximols (10.8 mg THC/10 mg CBD) or placebo, titrated up from one spray per day for the first 3 days to a maximum dose of four sprays/day	24 ineligible participants, placebo (n = 14) and Sativex (n = 15)	8 weeks (4 weeks of treatment + 4 weeks of observations)	to assess the feasibility and safety of nabiximols as a potential treatment for agitation in AD, defined by meeting four prespecified thresholds for recruitment, retention, adherence, and estimating a minimum effect size (≥0.3) on the Cohen–Mansfield Agitation Inventory (CMAI) score at Week 4	the clinical effect size for CMAI did not reach the desired threshold	[33]
Dementia	randomized, double-blind, placebo-controlled trial	“Avidekel,” cannabis oil (30% CBD and 1% THC: 295 mg and 12.5 mg per mL, respectively); 3 times a day	60 patients with a diagnosis of major neurocognitive disorder and associated behavioral disturbances; mean age: 79.4 years old; cannabis oil (n = 40); placebo (n = 20)	16 weeks	decrease, as compared to baseline, of four or more points on the CMAI score by week 16	positive	[34]
Parkinson’s Disease (PD)	double-blind randomized controlled trial	sublingual CBD-enriched product (101.9 mg/mL CBD, 4.8 mg/mL THC)	60 PD patients were randomized into CBD (n = 30) or placebo (n = 30)	12 weeks	CBD was safe (no adverse effects on motor, cognitive, or affective symptoms), improved Montreal Cognitive Assessment naming scores, but language scores increased in the placebo group, but remained unchanged in the CBD group	equivocal	[35]
PD	randomized trial	cannabis extract oral sesame oil solution increasing to a final dose of 2.5 mg/kg/day	PD patients with ≥20 on motor Movement Disorder Society Unified Parkinson’s Disease Rating Scale; CBD/THC (n = 31) or placebo (n = 30)	2 weeks	no benefit, worsened cognition and sleep, many mild adverse events, strong placebo response	negative	[36]
PD	randomized, double-blinded, placebo-controlled crossover clinical trial	purified CBD at a dose of 300 mg/day	24 individuals with PD, placebo, mean age: 64.13 years old	two experimental sessions within a 15-day interval	CBD attenuated the anxiety experimentally induced by the Simulated Public Speaking Test	positive	[37]
Essential tremor	randomized, controlled, double-blind, crossover study	single oral dose of purified CBD (300 mg) or placebo	19 patients, 10 M, 9 F, mean age: 63 years old	two experimental sessions were performed 2 weeks apart	no significant differences in upper limb tremors score, specific motor task tremor scores (writing and drawing/pouring) or clinical impression of change	negative	[38]
PD	exploratory double-blind trial	purified CBD 75 mg/day (n = 7), CBD 300 mg/day (n = 7) or placebo (n = 7)	21 PD patients without dementia or comorbid psychiatric conditions		no statistically significant differences in general symptoms score, plasma brain derived neurotrophic factor (BDNF) levels or magnetic resonance spectroscopy measures, CBD 300 mg/day had significantly different mean total scores in the well-being and quality of life	positive	[39]
Peripheral neuropathy	randomized and placebo-controlled trial	Oil: 250 mg CBD/3 fl. oz	29 patients with symptomatic peripheral neuropathy: CBD (n = 15) or placebo (n = 14)	4 weeks	reduction in intense pain, sharp pain, and cold and itchy sensations in the CBD group when compared to the placebo	positive	[40]
Chronic low back pain	randomized, placebo-controlled phase 3 trial	VER-01: a standardized full-spectrum extract from the *Cannabis sativa* L., each dose unit (2.5 mg THC, 0.1 mg cannabigerol and 0.02 mg CBD; sesame oil as excipient)	820 participants randomly assigned to VER-01 (n = 394) or placebo (n = 426)	2-week treatment phase (phase A), a 6-month open-label extension (phase B), followed by either a 6-month continuation (phase C) or randomized withdrawal (phase D)	reduced pain compared to the placebo group; the compound was also well-tolerated with no signs of dependence or withdrawal	positive	[41]

Abbreviations: AD—Alzheimer’s disease; BDNF—Brain-Derived Neurotrophic Factor; CBD—cannabidiol; CMAI—Cohen–Mansfield Agitation Inventory; DS—Dravet syndrome; F—female; LGS—Lennox–Gastaut syndrome; M—male; MAS—Modified Ashworth Scale; MS—multiple sclerosis; NRS—numerical rating scale; PD—Parkinson’s disease; THC—Delta-9-tetrahydrocannabinol.

## Data Availability

No new data were created or analyzed in this study. Data sharing is not applicable to this article.

## References

[1] Piao J.J., Kim S., Shin D., Lee H.J., Jeon K.H., Tian W.J., Hur K.J., Kang J.S., Park H.J., Cha J.Y. (2025). Cannabidiol alleviates chronic prostatitis and chronic pelvic pain syndrome via CB2 receptor activation and TRPV1 desensitization. World J. Mens Health.

[2] Zieba J., Sinclair D., Sebree T., Bonn-Miller M., Gutterman D., Siegel S., Karl T. (2019). Cannabidiol (CBD) reduces anxiety-related behavior in mice via an FMRP-independent mechanism. Pharmacol. Biochem. Behav..

[3] Chesworth R., Cheng D., Staub C., Karl T. (2022). Effect of long-term cannabidiol on learning and anxiety in a female Alzheimer’s disease mouse model. Front. Pharmacol..

[4] Liu Y.-M., Li J.-C., Gu Y.-F., Qiu R.-H., Huang J.-Y., Xue R., Li S., Zhang Y., Zhang K., Zhang Y.-Z. (2024). Cannabidiol exerts sedative and hypnotic effects in normal and insomnia model mice through activation of 5-HT1A receptor. Neurochem. Res..

[5] Sorkhou M., Bedder R.H., George T.P. (2021). The behavioral sequelae of cannabis use in healthy people: A systematic review. Front. Psychiatry.

[6] Cásedas G., de Yarza-Sancho M., López V. (2024). Cannabidiol (CBD): A systematic review of clinical and preclinical evidence in the treatment of pain. Pharmaceuticals.

[7] Pedrazzi J.F.C., Silva-Amaral D., Issy A.C., Gomes F.V., Crippa J.A., Guimarães F.S., Del Bel E. (2024). Cannabidiol attenuates prepulse inhibition disruption by facilitating TRPV1 and 5-HT1A receptor-mediated neurotransmission. Pharmacol. Biochem. Behav..

[8] Guldager M.B., Biojone C., da Silva N.R., Godoy L.D., Joca S. (2024). New insights into the involvement of serotonin and BDNF-TrkB signalling in cannabidiol’s antidepressant effect. Prog. Neuropsychopharmacol. Biol. Psychiatry.

[9] Annett S., Moore G., Robson T. (2020). FK506 binding proteins and inflammation related signalling pathways; basic biology, current status and future prospects for pharmacological intervention. Pharmacol. Ther..

[10] Romano S., Xiao Y., Nakaya M., D’Angelillo A., Chang M., Jin J., Hausch F., Masullo M., Feng X., Romano M.F. (2015). FKBP51 employs both scaffold and isomerase functions to promote NF-κB activation in melanoma. Nucleic Acids Res..

[11] Wang X., Lin C., Jin S., Wang Y., Peng Y., Wang X. (2023). Cannabidiol alleviates neuroinflammation and attenuates neuropathic pain via targeting FKBP5. Brain Behav. Immun..

[12] Cheng L., Xia F., Li Z., Shen C., Yang Z., Hou H., Sun S., Feng Y., Yong X., Tian X. (2023). Structure, function and drug discovery of GPCR signaling. Mol. Biomed..

[13] Perucca E., Bialer M. (2020). Critical aspects affecting cannabidiol oral bioavailability and metabolic elimination, and related clinical implications. CNS Drugs.

[14] Hossain K.R., Alghalayini A., Valenzuela S.M. (2023). Current challenges and opportunities for improved cannabidiol solubility. Int. J. Mol. Sci..

[15] Millar S.A., Stone N.L., Yates A.S., O’Sullivan S.E. (2018). A systematic review on the pharmacokinetics of cannabidiol in humans. Front. Pharmacol..

[16] Saals B.A.D.F., De Bie T.H., Osmanoglou E., van de Laar T., Tuin A.W., van Orten-Luiten A.C.B., Witkamp R.F. (2025). A high-fat meal significantly impacts the bioavailability and biphasic absorption of cannabidiol (CBD) from a CBD-rich extract in men and women. Sci. Rep..

[17] Beers J.L., Fu D., Jackson K.D. (2021). Cytochrome P450–catalyzed metabolism of cannabidiol to the active metabolite 7-hydroxy-cannabidiol. Drug Metab. Dispos..

[18] Madeo G., Kapoor A., Giorgetti R., Busardò F.P., Carlier J. (2023). Update on cannabidiol clinical toxicity and adverse effects: A systematic review. Curr. Neuropharmacol..

[19] Chesney E., Oliver D., Green A., Sovi S., Wilson J., Englund A., Freeman T.P., McGuire P. (2020). Adverse effects of cannabidiol: A systematic review and meta-analysis of randomized clinical trials. Neuropsychopharmacology.

[20] Huestis M.A., Solimini R., Pichini S., Pacifici R., Carlier J., Busardò F.P. (2019). Cannabidiol adverse effects and toxicity. Curr. Neuropharmacol..

[21] Sholler D.J., Schoene L., Spindle T.R. (2020). Therapeutic efficacy of cannabidiol (CBD): A review of the evidence from clinical trials and human laboratory studies. Curr. Addict. Rep..

[22] Devinsky O., Cross J.H., Laux L., Marsh E., Miller I., Nabbout R., Scheffer I.E., Thiele E.A., Wright S. (2017). Trial of cannabidiol for drug-resistant seizures in the Dravet syndrome. N. Engl. J. Med..

[23] Thiele E.A., Marsh E.D., French J.A., Mazurkiewicz-Beldzinska M., Benbadis S.R., Joshi C., Lyons P.D., Taylor A., Roberts C., Sommerville K. (2018). Cannabidiol in patients with seizures associated with Lennox–Gastaut syndrome (GWPCARE4): A randomised, double-blind, placebo-controlled phase 3 trial. Lancet.

[24] Devinsky O., Patel A.D., Cross J.H., Villanueva V., Wirrell E.C., Privitera M., Greenwood S.M., Roberts C., Checketts D., VanLandingham K.E. (2018). Effect of cannabidiol on drop seizures in the Lennox–Gastaut syndrome. N. Engl. J. Med..

[25] Thiele E., Marsh E., Mazurkiewicz-Beldzinska M., Halford J.J., Gunning B., Devinsky O., Checketts D., Roberts C. (2019). Cannabidiol in patients with Lennox–Gastaut syndrome: Interim analysis of an open-label extension study. Epilepsia.

[26] O’Brien T.J., Berkovic S.F., French J.A., Messenheimer J.A., Sebree T.B., Bonn-Miller M.O., Gutterman D.L. (2022). Adjunctive transdermal cannabidiol for adults with focal epilepsy. JAMA Netw. Open.

[27] Chen K.-A., Farrar M., Cardamone M., Gill D., Smith R., Cowell C.T., Truong L., Lawson J.A. (2018). Cannabidiol for treating drug-resistant epilepsy in children: The New South Wales experience. Med. J. Aust..

[28] Collin C., Davies P., Mutiboko I.K., Ratcliffe S., Sativex Spasticity in MS Study Group (2007). Randomized controlled trial of cannabis-based medicine in spasticity caused by multiple sclerosis. Eur. J. Neurol..

[29] Markovà J., Essner U., Akmaz B., Marinelli M., Trompke C., Lentschat A., Vila C. (2019). Sativex^®^ as add-on therapy vs. further optimized first-line antispastics (SAVANT) in resistant multiple sclerosis spasticity: A double-blind, placebo-controlled randomised clinical trial. Int. J. Neurosci..

[30] Bethoux F.A., Farrell R., Checketts D., Sahr N., Berwaerts J., Alexander J.K., Skobieranda F. (2024). A randomized, double-blind, placebo-controlled trial to evaluate the effect of nabiximols oromucosal spray on clinical measures of spasticity in patients with multiple sclerosis. Mult. Scler. Relat. Disord..

[31] Mousavi P., Emadzadeh M., Karimikhoshnoudian B., Sahraian M.A., Ghaffari M., Shaygannejad V., Payere M., Baghaei A., Zabeti A., Nahayati M. (2025). A randomized trial on efficacy of purified cannabidiol on spasticity in multiple sclerosis patients with gait problems: First report in Iran. Naunyn Schmiedebergs Arch. Pharmacol..

[32] Velayudhan L., Dugonjic M., Pisani S., Harborow L., Aarsland D., Bassett P., Bhattacharyya S. (2024). Cannabidiol for behavioral symptoms in Alzheimer’s disease (CANBiS-AD): A randomized, double-blind, placebo-controlled trial. Int. Psychogeriatr..

[33] Albertyn C.P., Guu T.-W., Chu P., Creese B., Young A., Velayudhan L., Bhattacharyya S., Jafari H., Kaur S., Kandangwa P. (2025). Sativex (nabiximols) for the treatment of agitation and aggression in Alzheimer’s dementia in UK nursing homes: A randomised, double-blind, placebo-controlled feasibility trial. Age Ageing.

[34] Hermush V., Ore L., Stern N., Mizrahi N., Fried M., Krivoshey M., Staghon E., Lederman V.E., Bar-Lev Schleider L. (2022). Effects of rich cannabidiol oil on behavioral disturbances in patients with dementia: A placebo-controlled randomized clinical trial. Front. Med..

[35] Mitarnun W., Kanjanarangsichai A., Junlaor P., Kongngern L., Mitarnun W., Pangwong W., Nonghan P. (2025). Cannabidiol and cognitive functions/inflammatory markers in Parkinson’s disease: A double-blind randomized controlled trial at Buriram Hospital (CBD-PD-BRH trial). Park. Relat. Disord..

[36] Liu Y., Bainbridge J., Sillau S., Rajkovic S., Adkins M., Domen C.H., Thompson J.A., Seawalt T., Klawitter J., Sempio C. (2024). Short-term cannabidiol with Δ-9-Tetrahydrocannabinol in Parkinson’s disease: A randomized trial. Mov. Disord..

[37] de Faria S.M., de Morais Fabrício D., Tumas V., Castro P.C., Ponti M.A., Hallak J.E., Zuardi A.W., Crippa J.A.S., Chagas M.H.N. (2020). Effects of acute cannabidiol administration on anxiety and tremors induced by a simulated public speaking test in patients with Parkinson’s disease. J. Psychopharmacol..

[38] Santos de Alencar S., Crippa J.A.S., Brito M.C.M., Pimentel Â.V., Cecilio Hallak J.E., Tumas V. (2021). A single oral dose of cannabidiol did not reduce upper limb tremor in patients with essential tremor. Park. Relat. Disord..

[39] Chagas M.H.N., Zuardi A.W., Tumas V., Pena-Pereira M.A., Sobreira E.T., Bergamaschi M.M., dos Santos A.C., Teixeira A.L., Hallak J.E.C., Crippa J.A.S. (2014). Effects of cannabidiol in the treatment of patients with Parkinson’s disease: An exploratory double-blind trial. J. Psychopharmacol..

[40] Xu D.H., Cullen B.D., Tang M., Fang Y. (2020). The effectiveness of topical cannabidiol oil in symptomatic relief of peripheral neuropathy of the lower extremities. Curr. Pharm. Biotechnol..

[41] Karst M., Meissner W., Sator S., Keßler J., Schoder V., Häuser W. (2025). Full-spectrum extract from *Cannabis sativa* DKJ127 for chronic low back pain: A phase 3 randomized placebo-controlled trial. Nat. Med..

[42] Fisher R.S., Acevedo C., Arzimanoglou A., Bogacz A., Cross J.H., Elger C.E., Engel J., Forsgren L., French J.A., Glynn M. (2014). ILAE official report: A practical clinical definition of epilepsy. Epilepsia.

[43] Ludányi A., Erőss L., Czirják S., Vajda J., Halász P., Watanabe M., Palkovits M., Maglóczky Z., Freund T.F., Katona I. (2008). Downregulation of the CB1 cannabinoid receptor and related molecular elements of the endocannabinoid system in epileptic human hippocampus. J. Neurosci..

[44] Maglóczky Z., Tóth K., Karlócai R., Nagy S., Erőss L., Czirják S., Vajda J., Rásonyi G., Kelemen A., Juhos V. (2010). Dynamic changes of CB1-receptor expression in hippocampi of epileptic mice and humans. Epilepsia.

[45] Fezza F., Marrone M.C., Avvisati R., Di Tommaso M., Lanuti M., Rapino C., Mercuri N.B., Maccarrone M., Marinelli S. (2014). Distinct modulation of the endocannabinoid system upon kainic acid-induced in vivo seizures and in vitro epileptiform bursting. Mol. Cell. Neurosci..

[46] Wallace M.J., Blair R.E., Falenski K.W., Martin B.R., DeLorenzo R.J. (2003). The endogenous cannabinoid system regulates seizure frequency and duration in a model of temporal lobe epilepsy. J. Pharmacol. Exp. Ther..

[47] Marsicano G., Goodenough S., Monory K., Hermann H., Eder M., Cannich A., Azad S.C., Cascio M.G., Gutiérrez S.O., Van der Stelt M. (2003). CB1 cannabinoid receptors and on-demand defense against excitotoxicity. Science.

[48] Sugaya Y., Yamazaki M., Uchigashima M., Kobayashi K., Watanabe M., Sakimura K., Kano M. (2016). Crucial roles of the endocannabinoid 2-arachidonoylglycerol in the suppression of epileptic seizures. Cell Rep..

[49] Reynolds J.R. (1862). Epilepsy: Its symptoms, treatment, and relation to other chronic convulsive diseases. Br. Foreign Medico-Chir. Rev..

[50] Epilepsy and Other Chronic Convulsive Diseases: Their Causes, Symptoms and Treatment. https://collections.nlm.nih.gov/catalog/nlm:nlmuid-100954847-bk.

[51] Chiu P., Olsen D.M., Borys H.K., Karler R., Turkanis S.A. (1979). The influence of cannabidiol and Δ^9^-tetrahydrocannabinol on cobalt epilepsy in rats. Epilepsia.

[52] Landucci E., Mazzantini C., Lana D., Calvani M., Magni G., Giovannini M.G., Pellegrini-Giampietro D.E. (2022). Cannabidiol inhibits microglia activation and mitigates neuronal damage induced by kainate in an in vitro seizure model. Neurobiol. Dis..

[53] Khan A.A., Shekh-Ahmad T., Khalil A., Walker M.C., Ali A.B. (2018). Cannabidiol exerts antiepileptic effects by restoring hippocampal interneuron functions in a temporal lobe epilepsy model. Br. J. Pharmacol..

[54] Jones N.A., Hill A.J., Smith I., Bevan S.A., Williams C.M., Whalley B.J., Stephens G.J. (2010). Cannabidiol displays antiepileptiform and antiseizure properties in vitro and in vivo. J. Pharmacol. Exp. Ther..

[55] Javadzadeh Y., Santos A., Aquilino M.S., Mylvaganam S., Urban K., Carlen P.L. (2024). Cannabidiol exerts anticonvulsant effects alone and in combination with Δ^9^-THC through the 5-HT1A receptor in the neocortex of mice. Cells.

[56] Martínez-Aguirre C., Márquez L.A., Santiago-Castañeda C.L., Carmona-Cruz F., Nuñez-Lumbreras M.d.l.A., Martínez-Rojas V.A., Alonso-Vanegas M., Aguado-Carrillo G., Gómez-Víquez N.L., Galván E.J. (2023). Cannabidiol modifies the glutamate over-release in brain tissue of patients and rats with epilepsy: A pilot study. Biomedicines.

[57] Martinez-Rojas V.A., Márquez L.A., Martinez-Aguirre C., Sollozo-Dupont I., López Preza F.I., Fuentes Mejía M., Alonso M., Rocha L., Galván E.J. (2025). Cannabidiol reduces synaptic strength and neuronal firing in layer V pyramidal neurons of the human cortex with drug-resistant epilepsy. Front. Pharmacol..

[58] Yu Y., Yang Z., Jin B., Qin X., Zhu X., Sun J., Huo L., Wang R., Shi Y., Jia Z. (2020). Cannabidiol inhibits febrile seizure by modulating AMPA receptor kinetics through its interaction with the N-terminal domain of GluA1/GluA2. Pharmacol. Res..

[59] Song H., Wang Y., Wang L., Guo C., Liu S., Rong Y., Tian J., Peng C., Shao Y., Ma Z. (2025). The DEC2–SCN2A axis is essential for the anticonvulsant effects of cannabidiol by modulating neuronal plasticity. Adv. Sci..

[60] Debski K.J., Ceglia N., Ghestem A., Ivanov A.I., Brancati G.E., Bröer S., Bot A.M., Müller J.A., Schoch S., Becker A. (2020). The circadian dynamics of the hippocampal transcriptome and proteome is altered in experimental temporal lobe epilepsy. Sci. Adv..

[61] Massey S., Quigley A., Rochfort S., Christodoulou J., Van Bergen N.J. (2024). Cannabinoids and Genetic Epilepsy Models: A Review with Focus on CDKL5 Deficiency Disorder. Int. J. Mol. Sci..

[62] Barker-Haliski M., Hawkins N.A. (2024). Innovative Drug Discovery Strategies in Epilepsy: Integrating Next-Generation Syndrome-Specific Mouse Models to Address Pharmacoresistance and Epileptogenesis. Expert Opin. Drug Discov..

[63] Yip K.L., Udoh M., Sharman L.A., Harman T., Bedoya-Pérez M., Anderson L.L., Banister S.D., Arnold J.C. (2025). Cannabinoid-like Compounds Found in Non-Cannabis Plants Exhibit Antiseizure Activity in Genetic Mouse Models of Drug-Resistant Epilepsy. Epilepsia.

[64] Ma L., Gao Y., Chen J., Hai D., Yu J., Tang S., Liu N., Liu Y. (2025). Cannabidiol Ameliorates Seizures and Neuronal Damage in Ferric Chloride-Induced Posttraumatic Epilepsy by Targeting TRPV1 Channel. J. Ethnopharmacol..

[65] Garcia-Cairasco N., Umeoka E.H.L., Cortes de Oliveira J.A. (2017). The Wistar Audiogenic Rat (WAR) Strain and Its Contributions to Epileptology and Related Comorbidities: History and Perspectives. Epilepsy Behav..

[66] Vitale R.M., Iannotti F.A., Amodeo P. (2021). The (Poly)Pharmacology of Cannabidiol in Neurological and Neuropsychiatric Disorders: Molecular Mechanisms and Targets. Int. J. Mol. Sci..

[67] Jones N.A., Glyn S.E., Akiyama S., Hill T.D.M., Hill A.J., Weston S.E., Burnett M.D.A., Yamasaki Y., Stephens G.J., Whalley B.J. (2012). Cannabidiol Exerts Anti-Convulsant Effects in Animal Models of Temporal Lobe and Partial Seizures. Seizure.

[68] Singh A., Madaan P., Bansal D. (2025). Update on Cannabidiol in Drug-Resistant Epilepsy. Indian J. Pediatr..

[69] Specchio N., Auvin S., Greco T., Lagae L., Nortvedt C., Zuberi S.M. (2025). Clinically Meaningful Reduction in Drop Seizures in Patients with Lennox–Gastaut Syndrome Treated with Cannabidiol: Post Hoc Analysis of Phase 3 Clinical Trials. CNS Drugs.

[70] Devinsky O., Patel A.D., Thiele E.A., Wong M.H., Appleton R., Harden C.L., Greenwood S., Morrison G., Sommerville K., GWPCARE1 Part A Study Group (2018). Randomized, Dose-Ranging Safety Trial of Cannabidiol in Dravet Syndrome. Neurology.

[71] Börnsen L., Romme Christensen J., Ratzer R., Hedegaard C., Søndergaard H.B., Krakauer M., Hesse D., Nielsen C.H., Sorensen P.S., Sellebjerg F. (2015). Endogenous Interferon-β-Inducible Gene Expression and Interferon-β Treatment Are Associated with Reduced T Cell Responses to Myelin Basic Protein in Multiple Sclerosis. PLoS ONE.

[72] Navarrete C., García-Martín A., Rolland A., DeMesa J., Muñoz E. (2021). Cannabidiol and Other Cannabinoids in Demyelinating Diseases. Int. J. Mol. Sci..

[73] Tomaszewska-Zaremba D., Gajewska A., Misztal T. (2025). Anti-Inflammatory Effects of Cannabinoids in Therapy of Neurodegenerative Disorders and Inflammatory Diseases of the CNS. Int. J. Mol. Sci..

[74] Fleisher-Berkovich S., Sharon N., Ventura Y., Feinshtein V., Gorelick J., Bernstein N., Ben-Shabat S. (2024). Selected Cannabis Cultivars Modulate Glial Activation: In Vitro and In Vivo Studies. J. Cannabis Res..

[75] Navarrete C., García-Martín A., Correa-Sáez A., Prados M.E., Fernández F., Pineda R., Mazzone M., Álvarez-Benito M., Calzado M.A., Muñoz E. (2022). A Cannabidiol Aminoquinone Derivative Activates the PP2A/B55α/HIF Pathway and Shows Protective Effects in a Murine Model of Traumatic Brain Injury. J. Neuroinflamm..

[76] Duncan R.S., Riordan S.M., Gernon M.C., Koulen P. (2024). Cannabinoids and Endocannabinoids as Therapeutics for Nervous System Disorders: Preclinical Models and Clinical Studies. Neural Regen. Res..

[77] Furgiuele A., Cosentino M., Ferrari M., Marino F. (2021). Immunomodulatory Potential of Cannabidiol in Multiple Sclerosis: A Systematic Review. J. Neuroimmune Pharmacol..

[78] Jones É., Vlachou S. (2020). A Critical Review of the Role of the Cannabinoid Compounds Δ9-Tetrahydrocannabinol (Δ9-THC) and Cannabidiol (CBD) and Their Combination in Multiple Sclerosis Treatment. Molecules.

[79] Elliott D.M., Singh N., Nagarkatti M., Nagarkatti P.S. (2018). Cannabidiol Attenuates Experimental Autoimmune Encephalomyelitis Model of Multiple Sclerosis through Induction of Myeloid-Derived Suppressor Cells. Front. Immunol..

[80] González-García C., Torres I.M., García-Hernández R., Campos-Ruíz L., Esparragoza L.R., Coronado M.J., Grande A.G., García-Merino A., Sánchez López A.J. (2017). Mechanisms of Action of Cannabidiol in Adoptively Transferred Experimental Autoimmune Encephalomyelitis. Exp. Neurol..

[81] Kozela E., Lev N., Kaushansky N., Eilam R., Rimmerman N., Levy R., Ben-Nun A., Juknat A., Vogel Z. (2011). Cannabidiol Inhibits Pathogenic T Cells, Decreases Spinal Microglial Activation and Ameliorates Multiple Sclerosis-like Disease in C57BL/6 Mice. Br. J. Pharmacol..

[82] Rahimi A., Faizi M., Talebi F., Noorbakhsh F., Kahrizi F., Naderi N. (2015). Interaction between the Protective Effects of Cannabidiol and Palmitoylethanolamide in an Experimental Model of Multiple Sclerosis in C57BL/6 Mice. Neuroscience.

[83] Akhavan Tavakoli M., Soleimani M., Marzban H., Shabani R., Moradi F., Ajdary M., Mehdizadeh M. (2023). Autophagic Molecular Alterations in the Mouse Cerebellum Experimental Autoimmune Encephalomyelitis Model Following Treatment with Cannabidiol and Fluoxetine. Mol. Neurobiol..

[84] Vitarelli da Silva T., Bernardes D., Oliveira-Lima O.C., Fernandes Pinto B., Limborço Filho M., Fraga Faraco C.C., Juliano M.A., Esteves Arantes R.M., Moreira F.A., Carvalho-Tavares J. (2024). Cannabidiol Attenuates In Vivo Leukocyte Recruitment to the Spinal Cord Microvasculature at Peak Disease of Experimental Autoimmune Encephalomyelitis. Cannabis Cannabinoid Res..

[85] Al-Ghezi Z.Z., Miranda K., Nagarkatti M., Nagarkatti P.S. (2019). Combination of Cannabinoids, Δ9-Tetrahydrocannabinol and Cannabidiol, Ameliorates Experimental Multiple Sclerosis by Suppressing Neuroinflammation through Regulation of miRNA-Mediated Signaling Pathways. Front. Immunol..

[86] Al-Ghezi Z.Z., Busbee P.B., Alghetaa H., Nagarkatti P.S., Nagarkatti M. (2019). Combination of Cannabinoids, Delta-9-Tetrahydrocannabinol (THC) and Cannabidiol (CBD), Mitigates Experimental Autoimmune Encephalomyelitis (EAE) by Altering the Gut Microbiome. Brain Behav. Immun..

[87] Barnes M.P. (2006). Sativex^®^: Clinical Efficacy and Tolerability in the Treatment of Symptoms of Multiple Sclerosis and Neuropathic Pain. Expert Opin. Pharmacother..

[88] Sastre-Garriga J., Vila C., Clissold S., Montalban X. (2011). THC and CBD Oromucosal Spray (Sativex^®^) in the Management of Spasticity Associated with Multiple Sclerosis. Expert Rev. Neurother..

[89] Vermersch P. (2011). Sativex^®^ (Tetrahydrocannabinol + Cannabidiol), an Endocannabinoid System Modulator: Basic Features and Main Clinical Data. Expert Rev. Neurother..

[90] Russo M., Calabrò R.S., Naro A., Sessa E., Rifici C., D’Aleo G., Leo A., De Luca R., Quartarone A., Bramanti P. (2015). Sativex in the Management of Multiple Sclerosis-Related Spasticity: Role of the Corticospinal Modulation. Neural Plast..

[91] Aragona M., Onesti E., Tomassini V., Conte A., Gupta S., Gilio F., Pantano P., Pozzilli C., Inghilleri M. (2009). Psychopathological and Cognitive Effects of Therapeutic Cannabinoids in Multiple Sclerosis: A Double-Blind, Placebo-Controlled, Crossover Study. Clin. Neuropharmacol..

[92] Vachová M., Novotná A., Mareš J., Taláb R., Fiedler J., Lauder H., Taylor L., Etges T., Wright S., Nováková I. (2013). A Multicentre, Double-Blind, Randomised, Parallel-Group, Placebo-Controlled Study of the Effect of Long-Term Sativex^®^ Treatment on Cognition and Mood in Patients with Spasticity Due to Multiple Sclerosis. J. Mult. Scler..

[93] Schoedel K.A., Chen N., Hilliard A., White L., Stott C., Russo E., Wright S., Guy G., Romach M.K., Sellers E.M. (2011). A Randomized, Double-Blind, Placebo-Controlled, Crossover Study to Evaluate the Subjective Abuse Potential and Cognitive Effects of Nabiximols Oromucosal Spray in Subjects with a History of Recreational Cannabis Use. Hum. Psychopharmacol..

[94] D’hooghe M., Willekens B., Delvaux V., D’haeseleer M., Guillaume D., Laureys G., Nagels G., Vanderdonckt P., Van Pesch V., Popescu V. (2021). Sativex^®^ (Nabiximols) Cannabinoid Oromucosal Spray in Patients with Resistant Multiple Sclerosis Spasticity: The Belgian Experience. BMC Neurol..

[95] Wade D. (2012). Evaluation of the Safety and Tolerability Profile of Sativex: Is It Reassuring Enough?. Expert Rev. Neurother..

[96] Medicines and Healthcare Products Regulatory Agency (2010). Sativex Oromucosal Spray.

[97] Toledano R.S., Akirav I. (2025). Cannabidiol Prevents Cognitive and Social Deficits in a Male Rat Model of Alzheimer’s Disease through CB1 Activation and Inflammation Modulation. Neuropsychopharmacology.

[98] Heppner F.L., Ransohoff R.M., Becher B. (2015). Immune Attack: The Role of Inflammation in Alzheimer Disease. Nat. Rev. Neurosci..

[99] Akiyama H., Barger S., Barnum S., Bradt B., Bauer J., Cole G.M., Cooper N.R., Eikelenboom P., Emmerling M., Fiebich B.L. (2000). Inflammation and Alzheimer’s Disease. Neurobiol. Aging.

[100] Cao S., Fisher D.W., Rodriguez G., Yu T., Dong H. (2021). Comparisons of Neuroinflammation, Microglial Activation, and Degeneration of the Locus Coeruleus–Norepinephrine System in APP/PS1 and Aging Mice. J. Neuroinflamm..

[101] Mello-Hortega J.V., de Oliveira C.S., de Araujo V.S., Furtado-Alle L., Tureck L.V., Souza R.L.R. (2025). Cannabidiol and Alzheimer Disease: A Comprehensive Review and In Silico Insights into Molecular Interactions. Eur. J. Neurosci..

[102] Jahn H. (2013). Memory Loss in Alzheimer’s Disease. Dialogues Clin. Neurosci..

[103] Raïch I., Lillo J., Rebassa J.B., Griñán-Ferré C., Bellver-Sanchis A., Reyes-Resina I., Franco R., Pallàs M., Navarro G. (2025). Cannabidiol as a Multifaceted Therapeutic Agent: Mitigating Alzheimer’s Disease Pathology and Enhancing Cognitive Function. Alzheimer’s Res. Ther..

[104] Salgado K.D.C.B., Nascimento R.G.F., Coelho P.J.F.N., Oliveira L.A.M., Nogueira K.O.P.C. (2024). Cannabidiol Protects Mouse Hippocampal Neurons from Neurotoxicity Induced by Amyloid β-Peptide25–35. Toxicol. In Vitro.

[105] Esposito G., De Filippis D., Maiuri M.C., De Stefano D., Carnuccio R., Iuvone T. (2006). Cannabidiol Inhibits Inducible Nitric Oxide Synthase Protein Expression and Nitric Oxide Production in Beta-Amyloid-Stimulated PC12 Neurons through p38 MAP Kinase and NF-κB Involvement. Neurosci. Lett..

[106] Alali S., Riazi G., Ashrafi-Kooshk M.R., Meknatkhah S., Ahmadian S., Hooshyari Ardakani M., Hosseinkhani B. (2021). Cannabidiol Inhibits Tau Aggregation In Vitro. Cells.

[107] Coles M., Watt G., Kreilaus F., Karl T. (2020). Medium-Dose Chronic Cannabidiol Treatment Reverses Object Recognition Memory Deficits of APPswe/PS1ΔE9 Transgenic Female Mice. Front. Pharmacol..

[108] Mahanta A.K., Chaulagain B., Gothwal A., Singh J. (2025). Engineered PLGA Nanoparticles for Brain-Targeted Codelivery of Cannabidiol and pApoE2 through the Intranasal Route for the Treatment of Alzheimer’s Disease. ACS Biomater. Sci. Eng..

[109] Mahanta A.K., Chaulagain B., Trivedi R., Singh J. (2024). Mannose-Functionalized Chitosan-Coated PLGA Nanoparticles for Brain-Targeted Codelivery of CBD and BDNF for the Treatment of Alzheimer’s Disease. ACS Chem. Neurosci..

[110] de Paula Faria D., Estessi de Souza L., Duran F.L.S., Buchpiguel C.A., Britto L.R., Crippa J.A.S., Filho G.B., Real C.C. (2022). Cannabidiol Treatment Improves Glucose Metabolism and Memory in a Streptozotocin-Induced Alzheimer’s Disease Rat Model: A Proof-of-Concept Study. Int. J. Mol. Sci..

[111] Goodland M.N., Banerjee S., Niehoff M.L., Young B.J., Macarthur H., Butler A.A., Morley J.E., Farr S.A. (2025). Cannabidiol Improves Learning and Memory Deficits and Alleviates Anxiety in 12-Month-Old SAMP8 Mice. PLoS ONE.

[112] Ma B.-Q., Jia J.-X., Wang H., Li S.-J., Yang Z.-J., Wang X.-X., Yan X.-S. (2024). Cannabidiol Improves the Cognitive Function of SAMP8 Alzheimer’s Disease Model Mice Involving the Microbiota–Gut–Brain Axis. J. Toxicol. Environ. Health A.

[113] Watt G., Shang K., Zieba J., Olaya J., Li H., Garner B., Karl T. (2020). Chronic Treatment with 50 mg/kg Cannabidiol Improves Cognition and Moderately Reduces Aβ40 Levels in 12-Month-Old Male AβPPswe/PS1ΔE9 Transgenic Mice. J. Alzheimer’s Dis..

[114] Mendez M.F. (2021). The Relationship between Anxiety and Alzheimer’s Disease. J. Alzheimer’s Dis. Rep..

[115] Khodadadi H., Salles É.L., Jarrahi A., Costigliola V., Khan M., Yu J.C., Morgan J.C., Hess D.C., Vaibhav K., Dhandapani K.M. (2021). Cannabidiol Ameliorates Cognitive Function via Regulation of IL-33 and TREM2 Upregulation in a Murine Model of Alzheimer’s Disease. J. Alzheimer’s Dis..

[116] Bishara M.A., Chum P.P., Miot F.E.L., Hooda A., Hartman R.E., Behringer E.J. (2025). Molecular Pathogenesis of Alzheimer’s Disease Onset in a Mouse Model: Effects of Cannabidiol Treatment. Front. Neurosci..

[117] Scuderi C., Steardo L., Esposito G. (2014). Cannabidiol Promotes Amyloid Precursor Protein Ubiquitination and Reduction of Beta Amyloid Expression in SH-SY5YAPP+ Cells through PPARγ Involvement. Phytother. Res..

[118] Drożak P., Skrobas U., Drożak M. (2022). Cannabidiol in the Treatment and Prevention of Alzheimer’s Disease—A Comprehensive Overview of In Vitro and In Vivo Studies. J. Educ. Health Sport.

[119] Aso E., Sánchez-Pla A., Vegas-Lozano E., Maldonado R., Ferrer I. (2015). Cannabis-Based Medicine Reduces Multiple Pathological Processes in AβPP/PS1 Mice. J. Alzheimer’s Dis..

[120] Cheng D., Spiro A.S., Jenner A.M., Garner B., Karl T. (2014). Long-Term Cannabidiol Treatment Prevents the Development of Social Recognition Memory Deficits in Alzheimer’s Disease Transgenic Mice. J. Alzheimer’s Dis..

[121] Hao F., Feng Y. (2021). Cannabidiol (CBD) Enhanced the Hippocampal Immune Response and Autophagy of APP/PS1 Alzheimer’s Mice Uncovered by RNA-Seq. Life Sci..

[122] Arnanz M.A., Ruiz de Martín Esteban S., Martínez Relimpio A.M., Rimmerman N., Tweezer Zaks N., Grande M.T., Romero J. (2024). Effects of Chronic, Low-Dose Cannabinoids, Cannabidiol, Delta-9-Tetrahydrocannabinol and a Combination of Both, on Amyloid Pathology in the 5xFAD Mouse Model of Alzheimer’s Disease. Cannabis Cannabinoid Res..

[123] Sánchez-Fernández N., Gómez-Acero L., Castañé A., Adell A., Campa L., Bonaventura J., Brito V., Ginés S., Queiróz F., Silva H. (2024). A Combination of Δ9-Tetrahydrocannabinol and Cannabidiol Modulates Glutamate Dynamics in the Hippocampus of an Animal Model of Alzheimer’s Disease. Neurotherapeutics.

[124] Aumer B., Rosa-Porto R., Coles M., Ulmer N., Watt G., Kielstein H., Karl T. (2025). Combination Treatment with Medium Dose THC and CBD Had No Therapeutic Effect in a Transgenic Mouse Model for Alzheimer’s Disease but Affected Other Domains Including Anxiety-Related Behaviours and Object Recognition Memory. Pharmacol. Biochem. Behav..

[125] Omotayo O.P., Lemmer Y., Mason S. (2024). A Narrative Review of the Therapeutic and Remedial Prospects of Cannabidiol with Emphasis on Neurological and Neuropsychiatric Disorders. J. Cannabis Res..

[126] Farkhondeh T., Khan H., Aschner M., Samini F., Pourbagher-Shahri A.M., Aramjoo H., Roshanravan B., Hoyte C., Mehrpour O., Samarghandian S. (2020). Impact of Cannabis-Based Medicine on Alzheimer’s Disease by Focusing on the Amyloid β-Modifications: A Systematic Study. CNS Neurol. Disord. Drug Targets.

[127] Bahji A., Meyyappan A.C., Hawken E.R. (2020). Cannabinoids for the Neuropsychiatric Symptoms of Dementia: A Systematic Review and Meta-Analysis. Can. J. Psychiatry.

[128] Broers B., Bianchi F. (2024). Cannabinoids for Behavioral Symptoms in Dementia: An Overview. Pharmacopsychiatry.

[129] Papadopoulou L., Alexandri F., Tsolaki A., Moraitou D., Konsta A., Tsolaki M. (2022). Neuropsychiatric Symptoms in Dementia. The Added Value of Cannabinoids. Are They a Safe and Effective Choice? Case Series with Cannabidiol 3%. Ann. Case Rep..

[130] Alexandri F., Papadopoulou L., Tsolaki A., Papantoniou G., Athanasiadis L., Tsolaki M. (2024). The Effect of Cannabidiol 3% on Neuropsychiatric Symptoms in Dementia—Six-Month Follow-Up. Clin. Gerontol..

[131] Navarro C.E., Pérez J.C. (2024). Treatment of Neuropsychiatric Symptoms in Alzheimer’s Disease with a Cannabis-Based Magistral Formulation: An Open-Label Prospective Cohort Study. Med. Cannabis Cannabinoids.

[132] Varshney K., Patel A., Ansari S., Shet P., Panag S.S. (2023). Cannabinoids in Treating Parkinson’s Disease Symptoms: A Systematic Review of Clinical Studies. Cannabis Cannabinoid Res..

[133] Esfandi A., Mehrafarin A., Kalateh Jari S., Naghdi Badi H., Larijani K. (2025). Cannabidiol Extracted from *Cannabis sativa* L. Shows Neuroprotective Impacts Against 6-OHDA-Induced Neurotoxicity via Nrf2 Signal Transduction Pathway. Iran. J. Pharm. Res..

[134] Giuliano C., Francavilla M., Ongari G., Petese A., Ghezzi C., Rossini N., Blandini F., Cerri S. (2021). Neuroprotective and Symptomatic Effects of Cannabidiol in an Animal Model of Parkinson’s Disease. Int. J. Mol. Sci..

[135] Crivelaro do Nascimento G., Ferrari D.P., Guimaraes F.S., Del Bel E.A., Bortolanza M., Ferreira-Junior N.C. (2020). Cannabidiol Increases the Nociceptive Threshold in a Preclinical Model of Parkinson’s Disease. Neuropharmacology.

[136] Lima A.C., Bioni V.S., Becegato M.S., Meier Y., Cunha D.M.G., Aguiar N.A., Gonçalves N., Peres F.F., Zuardi A.W., Hallak J.E.C. (2025). Preventive Beneficial Effects of Cannabidiol in a Reserpine-Induced Progressive Model of Parkinsonism. Front. Pharmacol..

[137] Santos J.C.C.D., Aquino P.E.A., Rebouças C.S.M., Sallem C.C., Guizardi M.P.P., Noleto F.M., Zampieri D.S., Ricardo N.M.P.S., Brito D.H.A., Silveira E.R. (2025). Neuroprotective Effects of a Cannabidiol Nanoemulsion in a Rotenone-Induced Rat Model of Parkinson’s Disease: Insights into the Gut–Brain Axis. Eur. J. Pharmacol..

[138] Nascimento G.C., Bálico G.G., de Mattos B.A., Dos-Santos-Pereira M., Oliveira I.G.C., Queiroz M.E.C., do Carmo Heck L., Navegantes L.C., Guimarães F.S., Del-Bel E. (2025). Cannabidiol Improves L-DOPA-Induced Dyskinesia and Modulates Neuroinflammation and the Endocannabinoid, Endovanilloid and Nitrergic Systems. Prog. Neuropsychopharmacol. Biol. Psychiatry.

[139] Lapmanee S., Bhubhanil S., Wongchitrat P., Charoenphon N., Inchan A., Ngernsutivorakul T., Dechbumroong P., Khongkow M., Namdee K. (2024). Assessing the Safety and Therapeutic Efficacy of Cannabidiol Lipid Nanoparticles in Alleviating Metabolic and Memory Impairments and Hippocampal Histopathological Changes in Diabetic Parkinson’s Rats. Pharmaceutics.

[140] Belardo C., Iannotta M., Boccella S., Rubino R.C., Ricciardi F., Infantino R., Pieretti G., Stella L., Paino S., Marabese I. (2019). Oral Cannabidiol Prevents Allodynia and Neurological Dysfunctions in a Mouse Model of Mild Traumatic Brain Injury. Front. Pharmacol..

[141] Meyer E., Rieder P., Gobbo D., Candido G., Scheller A., de Oliveira R.M.W., Kirchhoff F. (2022). Cannabidiol Exerts a Neuroprotective and Glia-Balancing Effect in the Subacute Phase of Stroke. Int. J. Mol. Sci..

[142] Xu B.-T., Li M.-F., Chen K.-C., Li X., Cai N.-B., Xu J.-P., Wang H.-T. (2023). Mitofusin-2 Mediates Cannabidiol-Induced Neuroprotection against Cerebral Ischemia in Rats. Acta Pharmacol. Sin..

[143] Chen K., Xu B., Xiao X., Long L., Zhao Q., Fang Z., Tu X., Wang J., Xu J., Wang H. (2024). Involvement of CKS1B in the Anti-Inflammatory Effects of Cannabidiol in Experimental Stroke Models. Exp. Neurol..

[144] Dong S., Zhao H., Nie M., Sha Z., Feng J., Liu M., Lv C., Chen Y., Jiang W., Yuan J. (2024). Cannabidiol Alleviates Neurological Deficits after Traumatic Brain Injury by Improving Intracranial Lymphatic Drainage. J. Neurotrauma.

[145] Santiago-Castañeda C., Huerta de la Cruz S., Martínez-Aguirre C., Orozco-Suárez S.A., Rocha L. (2022). Cannabidiol Reduces Short- and Long-Term High Glutamate Release after Severe Traumatic Brain Injury and Improves Functional Recovery. Pharmaceutics.

[146] Jesus C.H.A., Ferreira M.V., Gasparin A.T., Rosa E.S., Genaro K., Crippa J.A.S., Chichorro J.G., Cunha J.M. (2022). Cannabidiol Enhances the Antinociceptive Effects of Morphine and Attenuates Opioid-Induced Tolerance in the Chronic Constriction Injury Model. Behav. Brain Res..

[147] Abraham A.D., Leung E.J.Y., Wong B.A., Rivera Z.M.G., Kruse L.C., Clark J.J., Land B.B. (2020). Orally Consumed Cannabinoids Provide Long-Lasting Relief of Allodynia in a Mouse Model of Chronic Neuropathic Pain. Neuropsychopharmacology.

[148] Sepulveda D.E., Vrana K.E., Graziane N.M., Raup-Konsavage W.M. (2022). Combinations of Cannabidiol and Δ9-Tetrahydrocannabinol in Reducing Chemotherapeutic-Induced Neuropathic Pain. Biomedicines.

[149] Mitchell V.A., Harley J., Casey S.L., Vaughan A.C., Winters B.L., Vaughan C.W. (2021). Oral Efficacy of Δ9-Tetrahydrocannabinol and Cannabidiol in a Mouse Neuropathic Pain Model. Neuropharmacology.

[150] Foss J.D., Farkas D.J., Huynh L.M., Kinney W.A., Brenneman D.E., Ward S.J. (2021). Behavioural and Pharmacological Effects of Cannabidiol (CBD) and the Cannabidiol Analogue KLS-13019 in Mouse Models of Pain and Reinforcement. Br. J. Pharmacol..

[151] Dos Santos R., Veras F., Netto G., Elisei L., Sorgi C., Faccioli L., Galdino G. (2023). Cannabidiol Prevents Chemotherapy-Induced Neuropathic Pain by Modulating Spinal TLR4 via Endocannabinoid System Activation. J. Pharm. Pharmacol..

[152] Boccella S., Fusco A., Ricciardi F., Morace A.M., Bonsale R., Perrone M., Marabese I., De Gregorio D., Belardo C., Posa L. (2025). Acute Kappa Opioid Receptor Blocking Disrupts the Pro-Cognitive Effect of Cannabidiol in Neuropathic Rats. Neuropharmacology.

[153] Choi J., Li G., Stephens K.L., Timko M.P., DeGeorge B.R. (2025). The Use of Cannabinoids in the Treatment of Peripheral Neuropathy and Neuropathic Pain: A Systematic Review. J. Hand Surg..

[154] Vela J., Dreyer L., Petersen K.K., Arendt-Nielsen L., Duch K.S., Kristensen S. (2022). Cannabidiol Treatment in Hand Osteoarthritis and Psoriatic Arthritis: A Randomized, Double-Blind, Placebo-Controlled Trial. Pain.

[155] Zubcevic K., Petersen M., Bach F.W., Heinesen A., Enggaard T.P., Almdal T.P., Holbech J.V., Vase L., Jensen T.S., Hansen C.S. (2023). Oral Capsules of Tetra-Hydro-Cannabinol (THC), Cannabidiol (CBD) and Their Combination in Peripheral Neuropathic Pain Treatment. Eur. J. Pain.

[156] Sagredo O., Pazos M.R., Satta V., Ramos J.A., Pertwee R.G., Fernández-Ruiz J. (2011). Neuroprotective Effects of Phytocannabinoid-Based Medicines in Experimental Models of Huntington’s Disease. J. Neurosci. Res..

[157] Valdeolivas S., Sagredo O., Delgado M., Pozo M.A., Fernández-Ruiz J. (2017). Effects of a Sativex-Like Combination of Phytocannabinoids on Disease Progression in R6/2 Mice, an Experimental Model of Huntington’s Disease. Int. J. Mol. Sci..

[158] Consroe P., Laguna J., Allender J., Snider S., Stern L., Sandyk R., Kennedy K., Schram K. (1991). Controlled Clinical Trial of Cannabidiol in Huntington’s Disease. Pharmacol. Biochem. Behav..

[159] Elsaid S., Kloiber S., Le Foll B. (2019). Effects of Cannabidiol (CBD) in Neuropsychiatric Disorders: A Review of Pre-Clinical and Clinical Findings. Progress in Molecular Biology and Translational Science.

[160] López-Sendón Moreno J.L., García Caldentey J., Trigo Cubillo P., Ruiz Romero C., García Ribas G., Alonso Arias M.A.A., García de Yébenes M.J., Tolón R.M., Galve-Roperh I., Sagredo O. (2016). A Double-Blind, Randomized, Cross-Over, Placebo-Controlled, Pilot Trial with Sativex in Huntington’s Disease. J. Neurol..

[161] Hardy J., Greer R., Huggett G., Kearney A., Gurgenci T., Good P. (2023). Phase IIb Randomized, Placebo-Controlled, Dose-Escalating, Double-Blind Study of Cannabidiol Oil for the Relief of Symptoms in Advanced Cancer (MedCan1-CBD). J. Clin. Oncol..

[162] Hardy J.R., Greer R.M., Pelecanos A.M., Huggett G.E., Kearney A.M., Gurgenci T.H., Good P.D. (2025). Medicinal Cannabis for Symptom Control in Advanced Cancer: A Double-Blind, Placebo-Controlled, Randomised Clinical Trial of 1:1 Tetrahydrocannabinol and Cannabidiol. Support. Care Cancer.

[163] Haney M., Choo T.-H., Tiersten A., Levin F.R., Grassetti A., DeSilva N., Arout C.A., Martinez D. (2025). Oral Cannabis for Taxane-Induced Neuropathy: A Pilot Randomized Placebo-Controlled Study. Cannabis Cannabinoid Res..

[164] Winkler A., Meis A.C., Hermann C. (2025). Placebo Effects in a RCT Assessing 30 Days of Low Dose Cannabidiol (CBD) Treatment for Psychological Distress in Stressed Students at Risk for Depression. J. Cannabis Res..

[165] Zhekova R.M., Perry R.N., Spinella T.C., Dockrill K., Stewart S.H., Barrett S.P. (2024). The Impact of Cannabidiol Placebo on Responses to an Acute Stressor: A Replication and Proof-of-Concept Study. J. Psychopharmacol..

[166] Kocis P.T., Wadrose S., Wakefield R.L., Ahmed A., Calle R., Gajjar R., Vrana K.E. (2023). CANNabinoid Drug Interaction Review (CANN-DIRTM). Med. Cannabis Cannabinoids.

[167] Ho J.J.Y., Goh C., Leong C.S.A., Ng K.Y., Bakhtiar A. (2024). Evaluation of Potential Drug–Drug Interactions with Medical Cannabis. Clin. Transl. Sci..

[168] Nader F.D., Lopes L.P.N., Ramos-Silva A., Matheus M.E. (2025). Evidence of Potential Drug Interactions Between Cannabidiol and Other Drugs: A Scoping Review to Guide Pharmaceutical Care. Planta Med..

[169] Giacomini K.M., Huang S.-M., Tweedie D.J., Benet L.Z., Brouwer K.L.R., Chu X., Dahlin A., Evers R., Fischer V., International Transporter Consortium (2010). Membrane Transporters in Drug Development. Nat. Rev. Drug Discov..

[170] Zhu H.-J., Wang J.-S., Markowitz J.S., Donovan J.L., Gibson B.B., Gefroh H.A., Devane C.L. (2006). Characterization of P-Glycoprotein Inhibition by Major Cannabinoids from Marijuana. J. Pharmacol. Exp. Ther..

[171] Wang J.-S., Zhu H.-J., Markowitz J.S., Donovan J.L., DeVane C.L. (2006). Evaluation of Antipsychotic Drugs as Inhibitors of Multidrug Resistance Transporter P-Glycoprotein. Psychopharmacology.

[172] Geffrey A.L., Pollack S.F., Bruno P.L., Thiele E.A. (2015). Drug–Drug Interaction Between Clobazam and Cannabidiol in Children with Refractory Epilepsy. Epilepsia.

[173] Balachandran P., Elsohly M., Hill K.P. (2021). Cannabidiol Interactions with Medications, Illicit Substances, and Alcohol: A Comprehensive Review. J. Gen. Intern. Med..

[174] Coates S., Bardhi K., Prasad B., Lazarus P. (2024). Evaluation of the Drug–Drug Interaction Potential of Cannabidiol Against UGT2B7-Mediated Morphine Metabolism Using Physiologically Based Pharmacokinetic Modeling. Pharmaceutics.

[175] Campos M.G., China M., Cláudio M., Capinha M., Torres R., Oliveira S., Fortuna A. (2024). Drug–Cannabinoid Interactions in Selected Therapeutics for Symptoms Associated with Epilepsy, Autism Spectrum Disorder, Cancer, Multiple Sclerosis, and Pain. Pharmaceuticals.

[176] Etkins J., So G.C., Lu J.B.L., Koyama S., Gisch D.L., Melo Ferreira R., Cheng Y., McClara K., Jun J., Miller M. (2025). Genotype–Specific Safety and Pharmacokinetics of Cannabidiol in Healthy Volunteers. Clin. Transl. Sci..

